# Invariant visual object recognition and shape processing in rats

**DOI:** 10.1016/j.bbr.2014.12.053

**Published:** 2015-05-15

**Authors:** Davide Zoccolan

**Affiliations:** Visual Neuroscience Lab, International School for Advanced Studies (SISSA), 34136 Trieste, Italy

**Keywords:** Invariant recognition, Rat, Rodent, Shape processing, Pattern vision

## Abstract

Invariant visual object recognition is the ability to recognize visual objects despite the vastly different images that each object can project onto the retina during natural vision, depending on its position and size within the visual field, its orientation relative to the viewer, etc. Achieving invariant recognition represents such a formidable computational challenge that is often assumed to be a unique hallmark of primate vision. Historically, this has limited the invasive investigation of its neuronal underpinnings to monkey studies, in spite of the narrow range of experimental approaches that these animal models allow. Meanwhile, rodents have been largely neglected as models of object vision, because of the widespread belief that they are incapable of advanced visual processing. However, the powerful array of experimental tools that have been developed to dissect neuronal circuits in rodents has made these species very attractive to vision scientists too, promoting a new tide of studies that have started to systematically explore visual functions in rats and mice. Rats, in particular, have been the subjects of several behavioral studies, aimed at assessing how advanced object recognition and shape processing is in this species. Here, I review these recent investigations, as well as earlier studies of rat pattern vision, to provide an historical overview and a critical summary of the status of the knowledge about rat object vision. The picture emerging from this survey is very encouraging with regard to the possibility of using rats as complementary models to monkeys in the study of higher-level vision.

## Highlights

• Rats are capable of invariant visual object recognition.
• Rats spontaneously perceive different views of a visual object as similar to each other, that is as instances of the same object.
• Rats are capable of a multifeatural, shape-based visual processing strategy.
• Rats can learn complex, configural visual discriminations.
• Rats spontaneously process composite visual patterns according to perceptual grouping cues.

## 1 Introduction

The greatest challenge that any vision system (whether biological or artificial) has to face is the extraction of the behaviorally relevant content of visual scenes from the largely variable images collected, as an input, by the retina [1–3]. Within the domain of object recognition, this challenge manifests as the need of recognizing previously seen objects, despite the fact that any new encounter with these objects will often result in radically different retinal input images. The process of recognizing objects in spite of this substantial variation in their appearance is commonly known as *invariant recognition* or, more precisely, *transformation-tolerant recognition* [3–6]. Quite obviously, the problem of understanding how the brain achieves invariant recognition is tightly linked to the problem of understanding how the brain processes and represents the shape of visual objects. Although shape is not a well-defined concept, intuitively, shape features are those properties of the visual objects that more reliably allow distinguishing each object from any other one in the retinal input images. As such, global visual attributes that are not very diagnostic of object identity (because they are likely shared by many different objects), such as overall brightness, contrast, size, area, etc., are commonly considered as *lower-level* visual properties, rather than shape features (and a processing strategy relying on such features is commonly considered as a lower-level strategy). On the other hand, specific arrangements of local straight and curved boundaries, oriented edges and corners [7–10], as well as oriented and unoriented local contrast patterns [11–13] and image fragments [14,15] (i.e., attributes that more uniquely define visual objects) are typically viewed as proper, *higher-level* shape features (and a processing strategy relying on such features is commonly considered as a higher-level strategy). Bearing in mind that a rigorous (or agreed-upon) definition of shape feature complexity does not actually exist (e.g., see [16] for a discussion), one could tentatively define an *advanced* visual processing system as following – a system capable of extracting from the retinal input those higher-order (shape) features that are diagnostic of object identity across the many (unpredictable) image transformations each object undergoes during natural vision. Throughout the article, terms such as *lower-level properties*, *higher-level features* and *advanced processing strategy* will be used according to the definitions provided above.

These definitions make very explicit the challenge at the core of object vision – the requirement of maintaining a high degree of specificity for the features defining the identity of visual objects, while, at the same time, disregarding the huge variation in the appearance of such diagnostic features [17]. Computationally, balancing this trade-off between specificity and invariance makes the development of machine vision systems extraordinary challenging [4,5,17,18]. On the other hand, humans are capable of detecting and classifying objects out of tens of thousands of possibilities [19] within a fraction of a second [20,21], which implies that their visual system implements an extremely efficient and reliable machinery to process object information. Studies in nonhuman primates have revealed that such a processing is carried out along a hierarchy of visual cortical areas known as the *ventral visual pathway* (or *stream*) [4,6,22–25]. This pathway starts in primary visual cortex and culminates in the anterior part of the inferotemporal cortex, which conveys the most explicit representations of visual objects – that is, the representations that allow the easiest read-out of object identity (and generalization to identity-preserving image transformations) using simple linear decoders [16,26–28]. Unfortunately, the neuronal mechanisms underlying the formation of increasingly explicit representations of visual objects along the ventral stream are still poorly understood. This is likely a consequence of the extraordinary complexity of the primate visual system [29–31] and of the limited range of experimental manipulations that primate studies allow at the molecular, synaptic, and circuitry levels. This disproportion between the complexity of the neuronal architecture under investigation and the limited array of experimental approaches that are available to carry out such an investigation has recently led several vision scientists to explore the potential of rodents as models of visual functions. With regard to object recognition, whether rodents can actually serve as useful models to understand its neuronal basis crucially depends on how advanced their visual recognition behavior is. In the following, I will critically review most of the literature concerned with rat pattern vision, shape processing and visual object recognition, arguing that there is enough behavioral evidence to suggest that this species does embody some of the core mechanisms underlying invariant object recognition. As such, the rat and, by homology, the mouse (given the similarity between the visual systems of these species [32–35]) may represent a powerful complementary model to the nonhuman primate in the invasive investigation of the neuronal substrates of object vision.

## 2 Visual or not visual?

Rats and mice are the more widespread laboratory animal species, accounting for over 80% of all research animals used in the European Union [36]. Vision, on the other hand, is arguably one of the main research topics in neuroscience, targeted by disciplines as diverse as system, cognitive, computational, cellular and developmental neuroscience [37,38]. Given these premises, one could expect rodent-based studies to be at the forefront of vision research, but, historically, this has not been the case. In fact, until very recently, rodents have been largely overlooked by the vision science community, because their brains have been assumed to lack advanced visual processing machinery. Such an assumption rests on the observation that rats and mice are nocturnal/crepuscular species [36], have much lower visual acuity than primates (e.g., ∼1 cycle/deg in pigmented rats [39–46], compared to 30–60 cycles/deg in human and macaque fovea [47–53]), and make extensive use of other sensory modalities, such as touch [54–56] and smell [57–61], when exploring and interacting with their environment. In addition, neurons in rodent primary visual cortex, while displaying many of the tuning properties found in higher mammalian species (e.g., orientation tuning [62–74]), are not arranged into the functional cortical modules, such as the orientation columns [68,73,75], which are typical of non-human primates and small carnivores. Historically, this has confined the research on rodent vision to developmental studies of cortical plasticity mechanisms [76–80] (e.g., ocular dominance plasticity during the critical period) and behavioral/lesion studies of learning, familiarity and memory (e.g., the anatomical substrates of object recognition memory; for a review see [81–85]). In other words, the visual abilities of rats and mice have been the subject of investigation not because of an interest into rodent visual processing per se, but only insofar as these species provided an easier access to the neuronal/anatomical substrates of plasticity and memory, as compared to monkey and cat cortex.

At the same time, some of the most compelling, although indirect, evidence about the ability of rodents to extract behaviorally relevant information from the visual environment and perform non-trivial computations on the visual input, can be found in non-vision studies. In fact, rodents have been extensively studied for their impressive spatial navigation abilities, typically displayed when foraging over large ranges around their nests [86], also at dusk, when visual cues are available and can play a key role in their orienting behavior. Laboratory rats, in particular, have been found to make not only excellent use of visual cues [87–90], but to preferentially rely on such cues (over olfactory and auditory cues and path integration [91–93]), when tested in spatial navigation tasks. At the neuronal level, the most remarkable signature of such a strong dependence of rat spatial behavior on vision is the locking of hippocampal place fields to spatial visual cues, and their remapping when such cues are changed [94–99]. As pointed out in a recent review [1], although the visual processing implied in navigation cannot simply be equated to the one underlying object recognition, still it must engage similarly advanced mechanisms in terms of extraction of a scene's content (e.g., visual landmarks and their relative spatial location) from a largely viewpoint- and lighting-dependent retinal input.

### 2.1 The rediscovery of rodent vision

The notion that rodents can actually serve as useful models to study vision has only recently taken hold in the vision science community, with a new tide of studies that, over the past few years, have started to systematically explore visual functions in rats and mice. The turning point in the appreciation of rodent models by vision scientists can be ascribed to two main factors. The first is the ever growing array of powerful experimental tools (e.g., optogenetics [100–102] and in vivo two-photon imaging [75,103]) that have been developed to dissect neuronal circuits in rodents [104] and, while possible to apply in other species, are currently far less advanced in (e.g.) non-human primates. The second is the increasing appreciation for rodent cognitive abilities [105], which is making them attractive models to study cognitive functions as complex as perceptual decision-making [106,107], rule learning [108] and working memory [109]. While the access to genetic manipulation has inspired a considerable number of neuroanatomy [32,33], imaging [73,110,111] and electrophysiology [72,112–115] studies of mouse visual cortex (culminating with the endowment, by the Allen Institute for Brain Science, of a $300 million budget to a 10-year project to study the mouse visual system [116,117]), the possibility of testing highly manageable animal models in complex visual tasks, such as shape processing and invariant object recognition, has fostered the behavioral investigation of rat visual abilities [118–128]. Several reviews have summarized the most recent advances in the understanding of the mouse visual system [129–131]. In this report, I will focus instead on the mounting behavioral evidence about rat capability for advanced processing of visual shape/object information (Section 5). This discussion will be introduced by an historical overview of early investigations of rat vision (Section 3), up to more recent studies of shape processing and object recognition in this species (Section 4).

## 3 Early investigations of rat pattern vision

The first behavioral studies on rat vision date back to one century ago. Some of these early investigators found either very scarce [132] or no [133,134] evidence for pattern vision in albino rats, while some others found that white rats were capable of form discrimination (e.g., discrimination of upward vs. inverted triangles [135,136]). Reading this early debate about rat visual capabilities is very instructive, because many of the issues these early authors discussed are the same later studies also faced when testing rat visual behavior: (1) the choice of the rat strain (albino vs. pigmented); (2) the design of the testing apparatus (e.g., imposing, or not, a fixed viewing distance); (3) the possible contribution of other modalities (e.g., tactile and olfactory) in experiments where rats are allowed to approach physical shapes/objects; and (4) the confounds due to low-level visual properties, such as brightness and size of the stimuli. This debate was eventually settled in a series of seminal studies published by Karl Lashley [137,138] and other authors [139–145] in the 30s. Lashley, in particular, using an ingenious jumping stand apparatus, not only obtained the first behavioral measure of visual acuity in pigmented and albino rats [43] (later confirmed by many studies [39–42,44–46]), but also provided the most compelling evidence of his time about the ability of pigmented rats to successfully discriminate visual shapes [137,138].

### 3.1 Earliest evidence of invariant recognition and advanced shape processing in rats

**Fig. 1:**
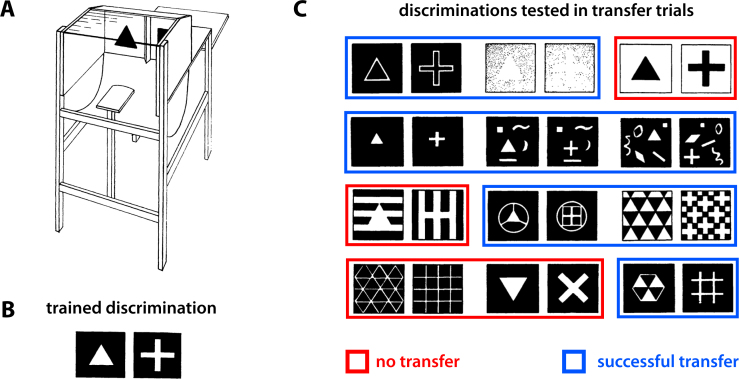
Extracts from Lashley's seminal study of rat pattern vision. (A) A sketch of the jumping stand apparatus introduced by Lashley to test rats in visual discrimination tasks (see Section 3.1 for a description). (B) An example of visual pattern discrimination, in which rats were trained in one of Lashley's experiments. (C) Alterations of the trained visual patterns (shown in B) to probe generalization of rat recognition in transfer trials. (For interpretation of the references to color in this figure legend, the reader is referred to the web version of this article.)

Modified from Lashley [138].

The jumping stand apparatus (shown in Fig. 1A) implemented a spatial two-alternative forced-choice procedure, with a rat required to jump against a target (positive) stimulus card from a distance of 20 cm, while ignoring a flanking negative stimulus card. The card bearing the negative stimulus was fixed rigidly, causing the rat to fall into a net for an incorrect jump toward it (punishment), while the card bearing the positive stimulus was held by a light spring, so that when a rat jumped against it, the card fell back and allowed the animal to reach a landing platform with food (reward). Using this approach, Lashley found that pigmented rats were able to discriminate different pairs of 2-dimensional geometrical shapes, such as an upward vs. an inverted triangle, a triangle vs. a circle, a triangle vs. a cross, etc. [137,138]. More importantly, he discovered that once an animal had learned to discriminate two shapes, he was able to generalize this discrimination “in spite of alteration in size, continuity of surface, or of outline [of the shapes and was] able to discover the figure in various combinations with irrelevant lines” [138] (see Fig. 1B and C). In addition, he also found that “marked changes in the luminous intensity of either figure or ground [did] not disturb reaction so long as the brightness relations of figure and ground [were] not reversed” [138] (e.g., see Fig. 1C, top row). These findings led Lashley to conclude that in rats “there must be some primitive generalization of form which goes beyond the recognition of identical elements” [138], which is what modern investigators would refer to as transformation-invariant or, more properly, transformation-tolerant visual object recognition (namely, size-tolerant, clutter-tolerant and luminance-tolerant recognition). On the other hand, Lashley found only limited tolerance to in-plane rotation (e.g., see Fig. 1C, central panel in the bottom row) and, by obscuring parts of the visual targets, he found “that the majority of animals [did] not react to the entire figure presented in the stimulus pattern. The lower or inner margin of the figures most frequently determine[d] the reaction. […] Animals react[ed] to such various characters as the distance of one figure from the frame, the relative surface areas of the figures, a conspicuous projecting point, and the like” [138]. At the same time, Lashley found that rats could learn to discriminate discontinuous oriented patterns made of distinct elements (e.g., two circles defining either a diagonal or a horizontal “line”) and that, more importantly, this discrimination generalized to continuous oriented lines with the same orientation of the previously learned discontinuous patterns. The reverse was also true: rats trained to discriminate between a vertical and a horizontal grating (i.e., patterns made of multiple, alternating black and white stripes, oriented either vertically or horizontally) generalized well not only to single continuous lines with the same orientation, but also to patterns made of single or multiple discontinuous lines with the same orientation (i.e., patterns made of either vertically or horizontally aligned arrays of small squares/rectangles). These findings induced Lashley to conclude that “the constants to which [a rat] reacts in otherwise variable situation are properties which can be derived only from a total figure, hence that his reaction is dependent upon some sort of unification of the elements within a part of the field” [138].

### 3.2 *Gestalt* visual perception in rats

These contrasting observations (i.e., the lack of “react[ion] to the entire [stimulus] figure” in some tests and the processing of “properties … derived … from a total figure” in some other tests [138]) anticipated contemporary attempts at determining the critical visual features underlying rat discrimination of visual patterns, and the ensuing debate about whether rats are actually capable of processing shape information across a large span of an object's surface or, instead, simply rely on local, lower-level image properties [120,121,146,147] (see Sections 4.2, 4.4 and 5.2). In the language of Lashley's time, these findings could be interpreted as evidence against or in favor of rat ability to process the total organization of visual patterns, by relying on the spontaneous grouping of sensory elements into whole form percepts, as postulated by Gestalt psychology. This topic was the subject of a series of studies by two of Lashley's contemporaries: Paul Field and Isadore Krechevsky, concerned, respectively, with the role of concept formation [140,141,145] and perceptual grouping [143,144] in rat visual perception.

**Fig. 2:**
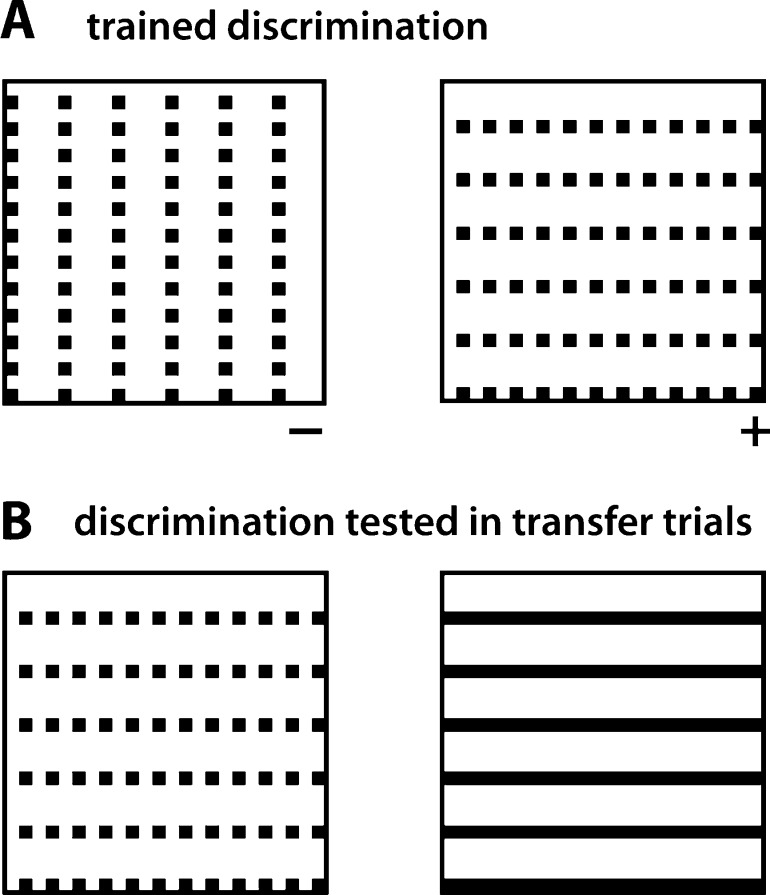
The visual patterns used by Krechevsky in his seminal study of perceptual grouping in rats. (A) Rats were initially trained to discriminate a negative (−) column pattern from a positive (+) row pattern. (B) When required to choose between the training-positive row pattern and a continuous horizontal grating, most rats consistently chose the latter.

Modified from Krechevsky [143,144].

In particular, Krechevsky [143,144] carried out a series of experiments in which, using Lashley's jumping stand, rats were trained to discriminate between two arrays made of the same number of identical constituent elements (small squares), whose proximity was larger along the vertical dimension in one array (thus forming a pattern of columns) and along the horizontal dimension in the other array (thus forming a pattern of rows). Not only did rats learn to perform this discrimination very effectively (thus showing a capability to process the spatial distribution of the constituent elements of the trained patterns), but, when tested in generalization trials, they showed a preference for a grating made of continuous lines with the same orientation of the originally learned training-positive array over the positive array itself. For instance, rats trained to respond to (i.e., jump toward) a pattern of rows (the positive stimulus; see Fig. 2A, right) and avoid a matching pattern of columns (the negative stimulus; see Fig. 2A, left), when presented with the same pattern of rows and a never-seen-before horizontal grating (see Fig. 2B), spontaneously chose the second over the first in the large majority of the transfer/generalization trials. Interestingly, such a preference for the continuous gratings was not observed in cases in which rats had been originally trained to discriminate a positive column (or row) pattern from a negative pattern made of a smaller number of elements, arranged so as to form an X [143]. Based on these findings, Krechevsky concluded that rat “perceptual process […] involves the operation of ‘forces of attraction’ between the members of a visual group of such a nature as to make the rat to prefer the continuous *gestalt* over that of the discontinuous groping even though the original training [was] on the discontinuous stimulus-complex” [143]. However, the emergence of such a preference for the continuous *gestalt* was not fully spontaneous, i.e., purely rooted into the “autochthonous forces of organization” [143] of the stimulus patterns, but depended on the need of the animal to extract such a *gestalt* to successfully accomplish the task. Hence, the failure of the continuous *gestalt* to emerge when the column/row patterns had to be discriminated from the X patterns, where a lower-level recognition strategy (e.g., one based on comparing the overall brightness of the patterns) would suffice to succeed in the task. Translated into contemporary terms, Krechevsky's findings (later confirmed by several studies [148–153]; see Section 4.3.2) can be considered as the first solid evidence of rat ability to integrate the constituent features of visual patterns into configural representations, up to the point of extracting from the training patterns abstract shape dimensions, such as verticalness and horizontalness. Perhaps more importantly, Krechevsky's interpretation of the impact of task complexity on the emergence of *gestalt* percepts anticipated the conclusions reached by the most recent studies of rat object recognition about the crucial role of task demands/constraints in determining the complexity of rat recognition strategy [118,120]. On the other hand, Krechevsky's conclusions are obviously limited by his lack of knowledge about the existence of orientation-selective filters in rat primary visual cortex (V1) [62–66,74]. In Section 4.3.2, I will discuss to what extent the tuning properties of V1 neurons can account for Krechevsky's findings, as well as for more recent studies of perceptual grouping in rats.

### 3.3 Strengths and limitations of early works on rat visual perception

Overall, the early studies by Lashley and his contemporaries indicate a capability of rats to use their vision to discriminate visual patterns and generalize to variations of previously learned discriminations (e.g., due to size changes of the target stimuli or addition of background clutter). These findings suggest that rats are able to, at least to some degree, abstract their recognition from the specific retinal input patterns produced by their encounters with visual objects. As such, Lashley's and related work can be considered as the first demonstration that rats are capable of a non-trivial processing strategy of visual patterns, i.e., a strategy that is not simply based on matching identical (or nearly identical) retinal inputs across consecutive encounters. However, one should be careful to interpret these findings as conclusive evidence for rat invariant object recognition.

In fact, Lashley's studies were affected by a lack of systematicity – although a large number of experiments were performed, with many different visual stimuli and manipulations, only a few rats per condition were tested and a small number of trials was collected per animal. In addition, only a small number of transformations of the trained visual patterns was tested, and across a limited variation range. Krechevsky's experiments were more focused, more thorough in terms of the number of tested rats and collected trials per animal, and, as such, statistically more rigorous. Still, Krechevsky's conclusions were also limited by the little variation of the patterns tested in his experiments (e.g., the spatial frequency and phase of the gratings were not varied). More critically, all these early studies based on Lashley's jumping stand were affected by two methodological issues that limited the scope of their conclusions, as far as invariant recognition is concerned. It is worth discussing these methodological limitations, since they can be found in many studies of rat vision, including some very recent ones [121,146].

#### 3.3.1 Limitations of spatial two-alternative forced-choice tasks

The major issue in Lashley's and related work is the use of a spatial two-alternative forced-choice procedure. By construction, this kind of task requires a subject to discriminate between two simultaneously presented visual patterns. As a consequence, discrimination can be based on the direct comparison of lower-level visual attributes (e.g., local luminance or contrast), whose relative magnitude is, generally, preserved across transformations, especially when both patterns are equally transformed, as in the case of Lashley's study. As an example, rat discrimination of a triangle from a square could be based on comparing the relative luminance of the bottom halves of these patterns, without necessarily processing any actual shape attribute (e.g., orientation of edges, presence and sharpness of corners, etc.). If these patterns are equally scaled or translated, or their overall luminosity is equally reduced/increased, the rat will still correctly discriminate them, since the relative brightness of their bottom halves will remain unchanged (the square's bottom half will always be brighter than the triangle's bottom half), de facto acting as a transformation-invariant, low-level, diagnostic image feature. Such a recognition strategy, while definitely more sophisticated than a simple matching of retinal inputs to fixed templates, should anyway not be considered as an advanced processing strategy, because it would not engage higher-order, transformation-tolerant representations of shape features. Lashley's work, as well as other rat vision studies based on two-alternative forced-choice tasks with identically transformed stimuli [121,146], cannot differentiate between such a low-level strategy and a truly invariant, shape-based processing of visual objects. This does not mean that, in general, two-alternative forced-choice procedures cannot be used to study shape processing (see, as an example, Simpson's and Gaffan's work [147], discussed in Section 4.2). Also invariant recognition can, in principle, be tested using these procedures, provided that the stimuli shown to the animals are independently transformed in each trial. However, this is very impractical, because of the combinatorial explosion that would result from building every possible pair of transformed stimuli (especially when the stimuli are transformed along many different dimensions – size, position, in-plane rotation, etc.). As shown in Section 5, a more practical and effective solution is to use a task where a single, isolated stimulus is presented in each trial [118–120].

#### 3.3.2 Limitations in the test of generalization

Another issue, when probing invariant object recognition in animal subjects, is to test generalization of recognition to novel views of previously learned objects, without giving any feedback about the identity of these views (by means of reward and/or punishment). This is obviously very challenging, because, ideally, it would require an animal to perform large numbers of unrewarded trials. However impractical, failure to withhold feedback implies an inability to test pure generalization of recognition, since the tested animals could, in principle, learn and memorize the correct association between new object views and reward, without necessarily perceiving such new views as similar to the ones they originally learned. In other words, a set of perceptually unrelated (for the animals) object views could be arbitrarily associated, in memory, to the same category, only because of the continuous training received during the task. Lashley's approach to address this issue, so as to test generalization of pattern discrimination, was actually well designed, given the technological limits of his time. After training a rat to discriminate a pair of visual patterns (using reward and punishment as described above), the animal was tested in a series of “critical” (i.e., generalization) trials, in which he was “allowed a free choice between [transformed versions of the patterns], receiving food at every trial, regardless of the pattern chosen” [138]. Using these punishment-free trials is obviously a good approximation to a pure test of generalization, yet is not completely robust, as acknowledged by Lashely himself [138] and Krechevsky [143]. Since an animal receives reward no matter what stimulus he chooses, he could, in principle, learn to consistently respond to (i.e., jump toward) a novel test pattern, based on the positive feedback he receives on the first (or the first few) trials. That is, a rat that happens to respond correctly on the first trial, purely by chance, would be reinforced in his initial (random) choice by the reward he receives. On the other hand, as reported by Lashley, an animal responding randomly, when initially confronted with two novel patterns, could learn “that no choice is necessary when new figures are presented and promptly [adopt] a position habit […] even with figures which in earlier tests gave perfect transfer” [138]. These shortcomings do not fully undermine the validity of Lashley's transfer tests, but could have led to either an overestimation or an underestimation of the generalization ability of rats that were, respectively, either initially successful or unsuccessful with new test patterns. More in general, the approach of testing generalization by rewarding whatever choice an animal makes on novel stimuli, while not perfect, is acceptable, as long as data from multiple animals are pooled to obtain group average performances (in which possible overestimations and underestimations are averaged out). Nevertheless, as shown in Section 5, novel approaches have been recently proposed that allow a cleaner assessment of generalization [118,119].

## 4 Pattern vision and visual shape processing in rats

The encouraging findings about rat visual processing abilities provided by Lashley and his contemporaries in the 30s did not succeed in triggering a sustained interest in this species (or any other rodent species) among vision scientists. In the ensuing decades, the vision science community became progressively more interested in monkeys and small carnivores (e.g., cats) as models to study the visual system, and virtually abandoned any systematic attempt at studying visual processing in rodents. Still, even before the very recent surge of interest in rodent vision (see Section 5), a few behavioral studies further explored the perceptual strategies underlying rat pattern vision and object recognition. Among these studies, the most noteworthy are those published by Sutherland and colleagues in the 60s [154–157] and by Gaffan and colleagues in the 90s [147,158–160].

### 4.1 In search of the critical stimulus dimensions underlying rat pattern vision

Sutherland developed a new apparatus to train rats in visual discrimination tasks [154–156]. It consisted in attaching to the rear wall of an operant box two shapes cut out of white Perspex (the discriminanda), with a drinking nozzle for reward delivery protruding through the middle of each shape. Rats were trained to leave a starting box (located at the opposite end of the wall bearing the discriminanda) and approach a fixed positive pattern (while ignoring the other negative pattern) to collect reward. Using this apparatus, Sutherland successfully trained pigmented rats to discriminate between a vertical and a horizontal rectangle [154]. More importantly, he also ran transfer (i.e., generalization) tests in which rats were presented with altered versions of the learned patterns and were rewarded independently of their choice (i.e., same approach to test generalization as Lashley's; see Section 3.3.2). These tests confirmed and extended Lashley's early reports about rat capability to discriminate visual shapes in spite of size variation (i.e., both larger and smaller versions of the rectangles) and changes to their surface/outline (i.e., breaking the rectangles into discontinuous segments).

In another series of studies [155,156], Sutherland and coworkers tried to infer the critical shape dimensions underlying rat discrimination of 2-dimensional visual patterns. They initially trained rats to discriminate between an “open” and a “close” shape (respectively, a diamond-like and a hourglass-like silhouette, either horizontally or vertically oriented), and then measured rat perceived similarity between each of 20 new transfer shapes and the originally trained shapes. These transfer tests were run not only using the spatial two-alternative forced-choice task described above, but also by presenting a single shape at the time in the wall bearing the discriminanda (leaving the other stimulus slot in the wall empty, with just the drinking nozzle protruding from the bare wall). Again, being these transfer tests, rats were rewarded no matter whether they chose to approach the nozzle protruding from the test shape or from the empty slot. Noticeably, this is one of the earliest attempts to overcome the limitations of two-alternative forced-choice procedures (see discussion in Section 3.3.1), so as to allow a more reliable assessment of the perceptual similarity between the transfer shapes and the originally learned diamond-like and hourglass-like patterns. The shapes that rats perceived as being more similar to the diamond-like pattern were those that, qualitatively, looked more as “closed” (i.e., more compact and without protruding elements), such as circles, ellipses, triangles and squares. By contrast, the shapes that rats perceived as being more similar to the hourglass-looking pattern were those that looked more “open” (i.e., more spread-out, with a lot of contour and projections), such as crosses, Hs, Us, etc. As a way to quantify this tendency of rats to rank and classify the transfer shapes along a “closeness–openness” stimulus dimension, the authors computed the ratio between contour length and square root of the area for each shape and found a strong, positive correlation between the ranking of the shapes according to their perceived similarity to the diamond-like (“closed”) shape and their ranking according to this ratio. Since this ratio provides a way to quantify the complexity (in terms of openness/closeness, i.e., presence or absence of protruding features) of 2-dimensional patterns (with more complex, spread-out shapes having a longer contour than simpler, more compact shapes, given the same area), Sutherland's and coauthors’ findings suggest a capability of rats to abstract their recognition from the specific retinal input patterns they were originally exposed to, so as to build quite explicit representations of abstract shape dimensions. This conclusion is in agreement with Krechevsky's [143,144] and related studies [148–153] about rat ability to learn the diagnostic dimensions of verticalness and horizontalness from discontinuous oriented patterns by means of perceptual grouping (see Sections 3.2 and 4.3.2), and with a more recent study [123] about rat capability of categorizing natural images on the base of global stimulus dimensions, such as convexity and aspect ratio.

Overall, Sutherland's studies suggest that the rat visual system is capable of the kind of advanced shape processing that is the hallmark of high-level vision: the extrapolation of abstract shape dimensions. However, once again, care should be taken before considering these findings as conclusive evidence for invariant object recognition in rats, because of several limitations in Sutherland's and coworkers’ experimental approach (mostly pointed out by the authors themselves). First, only few shapes were tested and very few trials were collected per shape (10 trials, in the transfer experiment with single-shape presentation), thus greatly limiting the accuracy of the measurement of rat recognition performance and, therefore, of rat perceived similarity between transfer and trained shapes. In addition, the single-shape presentation devised by Sutherland, while overcoming some of the issues concerning two-alternative forced-choice tasks (see Section 3.3.1), was itself affected by one main limitation: the rats showed a consistent bias toward the side on which the shape was presented (no matter whether they were supposed to approach it or avoid it), thus further reducing the power to estimate rat preference/perceived similarity [156]. More critically, it is difficult to understand to what extent other lower-level stimulus properties may have played a role in determining rat similarity judgments. Many different feature dimensions could, potentially, correlate with the shape–complexity ratio computed in [156]: shape area, average luminosity, overall luminosity, luminosity/contrast in the bottom/top halves of the shapes, etc. As shown in Section 5.2, classification image methods are arguably the best approaches to uncover what visual features a rat relies upon when performing an object discrimination task and, therefore, reveal the nature and complexity of rat recognition strategy [120,121]. On the other hand, ensuring that rats do not trivially discriminate visual objects because of differences in low-level properties can only be achieved in two ways: either by testing rats with a very large battery of object transformations or by carefully matching as many different low-level visual attributes as possible. While the former approach was applied in the most recent studies of rat invariant recognition [118–120], the latter was followed by Simpson and Gaffan in their very thorough investigation of visual scene and object processing in rats [147].

### 4.2 Evidence of shape-based visual object recognition in rats

**Fig. 3:**
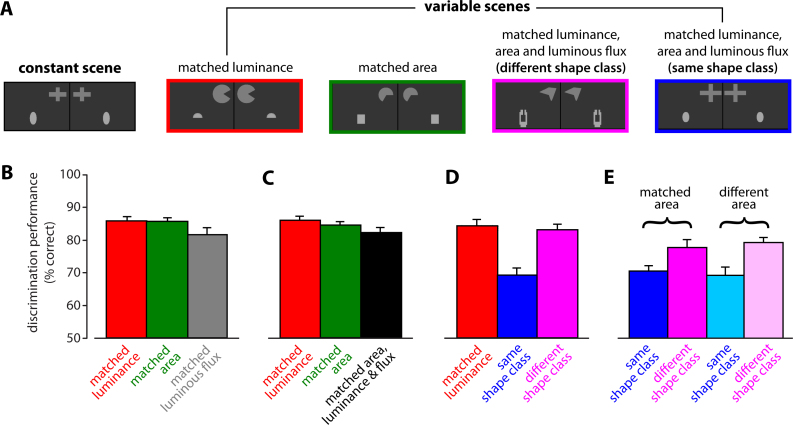
Summary of Simpson's and Gaffan's experimental design and results. (A) Examples of stimulus scenes used to probe rat pattern vision in Simpson's and Gaffan's study. In each trial, rats were rewarded for avoiding a negative-constant scene (right panel) and approaching a trial-specific variable scene (examples shown in the left panels). The objects in the variable scenes could be matched to those in the constant scene with respect to different visual properties: luminance, area, luminous flux (i.e., area × luminance), and shape class. (B–E) Rat recognition performance for different kinds of tested variable scenes. The color of the bars matches the color of the corresponding example variable scenes in (A). See Section 4.2 for a detailed description. (For interpretation of the references to color in this figure legend, the reader is referred to the web version of this article.)

Modified from Simpson and Gaffan [147].

Simpson's and Gaffan's study [147] was based on a procedure/apparatus to test rats in visual tasks that Gaffan and colleagues had described in previous reports [158,159], and that they later applied in a series of lesion studies to identify the neuronal substrates of visual object discrimination and memory in rats [160–164]. The apparatus consisted of a Y maze, with the three arms having equal length and the three angles formed by each arm pair having equal amplitude. A pair of computer monitors (jointed by a 117° angle, so as to subtend ∼94° horizontally, when viewed from the maze center) was located at the end of each arm, where reward was also delivered. Visual scenes of various complexity (containing a number of 2-dimensional objects, such as ellipses, ASCII text characters, polygons, rectangles, etc.) were displayed on the monitors, with the scenes shown in the adjacent monitors at the end of an arm being the mirror version of each other along the vertical axis (each pair of mirror scenes on adjacent monitors constituted one stimulus scene; see examples in Fig. 3A). Each rat was trained in a spatial two-alternative forced-choice task, in which two different stimulus scenes were displayed at the end of the two arms not currently occupied by the animal, and the rat had to learn to either approach a target positive-constant or avoid a target negative-constant scene. This constant scene was paired, in every trial, to a trial-unique variable scene, containing visual objects that could differ from those of the constant scene in a variety of properties (examples of a constant scene and some variable scenes that were paired to it in Simpson's and Gaffan's study [147] are shown in Fig. 3A).

Using this approach, Simpson and Gaffan showed that rats were able to discriminate pairs of stimulus scenes containing two visual objects per monitor, even when these objects occupied the same location within the visual space (i.e., within each scene), thus concluding that rats “must encode more than just the gross spatial distribution of patches (objects) that contrast with the background” [147]. More importantly, they found that rats maintained a high discrimination performance (above 80% correct) no matter whether position-matched objects in the stimulus scenes were approximately equated in terms of area, luminance (i.e., gray level, since each object was rendered in a uniform gray intensity value) and luminous flux (i.e., area × luminance). These three low-level features were matched independently in separate tests, thus showing that none of them was critical, by itself, for the successful discrimination of the visual scenes (compare bars in Fig. 3B). When all these properties were simultaneously matched (thus producing stimulus scenes containing objects with similar area, luminance and luminous flux) rats still successfully discriminated the scenes (compare bars in Fig. 3C), thus suggesting that they based their recognition on shape information. This conclusion was confirmed by requiring rats to discriminate scenes containing objects of the same shape category in matching visual field locations (e.g., ellipses with different aspect ratio and/or slight size differences; compare the constant scene to the last variable scene in Fig. 3A). This produced a drop in rat performance (see middle bar in Fig. 3D) that led Gaffan and Simpson to conclude “first, that rats perceived the objects as distinct shapes, not as luminous blobs, and second, that they encoded them in terms of visual features that are common to a class of shape” [147]. In addition, when rats were given the chance to base their discrimination on either shape differences or size differences among the visual objects contained in a pair of stimulus scenes, they appeared to rely only on the former (higher-level) property, while ignoring the latter (lower-level) attribute. That is, rat performance was equally good when the objects differed in shape (no matter whether they also differed in size; see purple bars in Fig. 3E), and equally worse when the objects had similar shape (no matter whether size differences were also present that could be used as low-level cues; see blue bars in Fig. 3E).

Overall, Gaffan's and Simpson's study provides compelling evidence about the capability of rats to discriminate visual objects on the base of shape differences. Although no attempt was done to infer what shape features the animals relied upon (as done by Sutherland and colleagues [156] and by more recent studies [120,121,123]), the systematic (although partial) matching of various low-level properties convincingly shows that rats can recognize visual patterns without resorting to gross differences in area, luminosity and luminous flux. In addition, the independence of rat performance on the area/size of the objects can be taken as a further demonstration of the size tolerance of rat object recognition. However, tolerance to parametric object variations along different transformation axes was not systematically investigated, thus leaving open the question of how much variability in object appearance, and along what dimensions, rats are able to tolerate. Finally, the experiments of Gaffan and Simpson were still affected by the limitations inherent in spatial two-alternative forced-choice tasks (see Section 3.3.1), although to a lesser extent than other studies, because of the large variety of shapes against which each learned target object had to be compared (e.g., compare the objects in the constant scene to those in the variable scenes of Fig. 3A), which made it more unlikely for the rats to rely on local low-level cues (e.g., luminance differences in the top or bottom halves of the objects, as later reported by Minini and Jeffery [146]; see Section 4.4).

Gaffan and colleagues further investigated rat visual object recognition in a series of lesion studies aimed at assessing the role of perirhinal cortex in object recognition memory and object identification [160–164]. Given that this review is mainly concerned with behavioral investigations of rat visual object recognition, I will not discuss the neuroanatomical implications of these studies (for a review, see [81–85]). Still, leaving aside the neuroanatomical findings, some of these studies provide additional behavioral evidence about rat visual processing abilities. For instance, in [160], rats were tested in a highly demanding task, which required them to discriminate the constant scene from variable scenes that differed from the constant in four possible ways: (1) both identity and position of the objects within the scenes; (2) only object identity (same position); (3) only object position (same identity); and (4) arrangement of objects with fixed identity across fixed positions. Since these four types of scene comparisons were randomly interleaved, the fact that control (i.e., non-lesioned) animals achieved an average performance of ∼70% correct (or greater) in each comparison suggests that rats are capable of a highly flexible recognition strategy, in which position and shape cues can be interchangeably extracted, depending on task demands [160].

### 4.3 Spatial integration of visual information in rats

Many other lesion studies of rat recognition memory have reported data about object discrimination behavior in this species. Unfortunately, most of these studies have used solid objects that rats were free to approach and touch, rather than purely visual stimuli (e.g., see [165–167]). In addition, recognition performance has been typically measured in terms of the time spent by an animal exploring a given object, rather than as the fraction of explicit correct identifications he made. This makes extremely hard to infer anything about rat visual processing, because, under such experimental settings, not only the visual properties of the objects (e.g., size, brightness, etc.) are not controlled, but rats can inspect the discriminanda using a mixture of visual, tactile and olfactory cues [168,169]. More recently, several investigators have developed approaches that, similarly to the one devised by Gaffan and colleagues, allow testing rats in purely visual tasks. These approaches are based either on V-shaped water mazes equipped with monitors for stimulus presentation [170–172] or on operant boxes equipped with touchscreens [146,173–177]. Still, most of these studies are not much concerned with rat visual processing per se, but, rather, rat visual behavior is exploited as a mean to test and understand learning and memory functions. As such, these studies, while providing additional evidence of rat capability to use vision to extract task-relevant information from visual patterns, do not typically include manipulations of the visual stimuli that allow inferring how advanced rat object vision is in terms of shape processing and invariant recognition.

#### 4.3.1 Configural visual discrimination in rats

Exceptions to this rule are a few reports about the ability of rats to learn complex configural visual discriminations [161,171,172], that is discriminations that require the concurrent processing of multiple features (or parts) of the visual stimuli. The most widely applied of such tasks is the biconditional discrimination, in which four different visual features (i.e., features A, B, C and D) are combined to obtain four different compound stimuli (i.e., compound patterns AB, AC, BC, and CD) that can only be distinguished from each other by concurrently considering both constituent elements of each pattern. While in monkey studies the biconditional discrimination is typically implemented by presenting one compound patterns at the time and then asking the monkey subject to report its identity by means of saccades or lever responses [178,179], in the context of rat studies this paradigm has been implemented in the form of multiple two-alternative forced-choice tasks (i.e., AB+ vs. AC−, DC+ vs. DB−, AB+ vs. DB− and DC+ vs. AC−, with the + denoting the rewarded pattern), administered to the animals in interleaved trials or small blocks of trials [161,172].

**Fig. 4:**
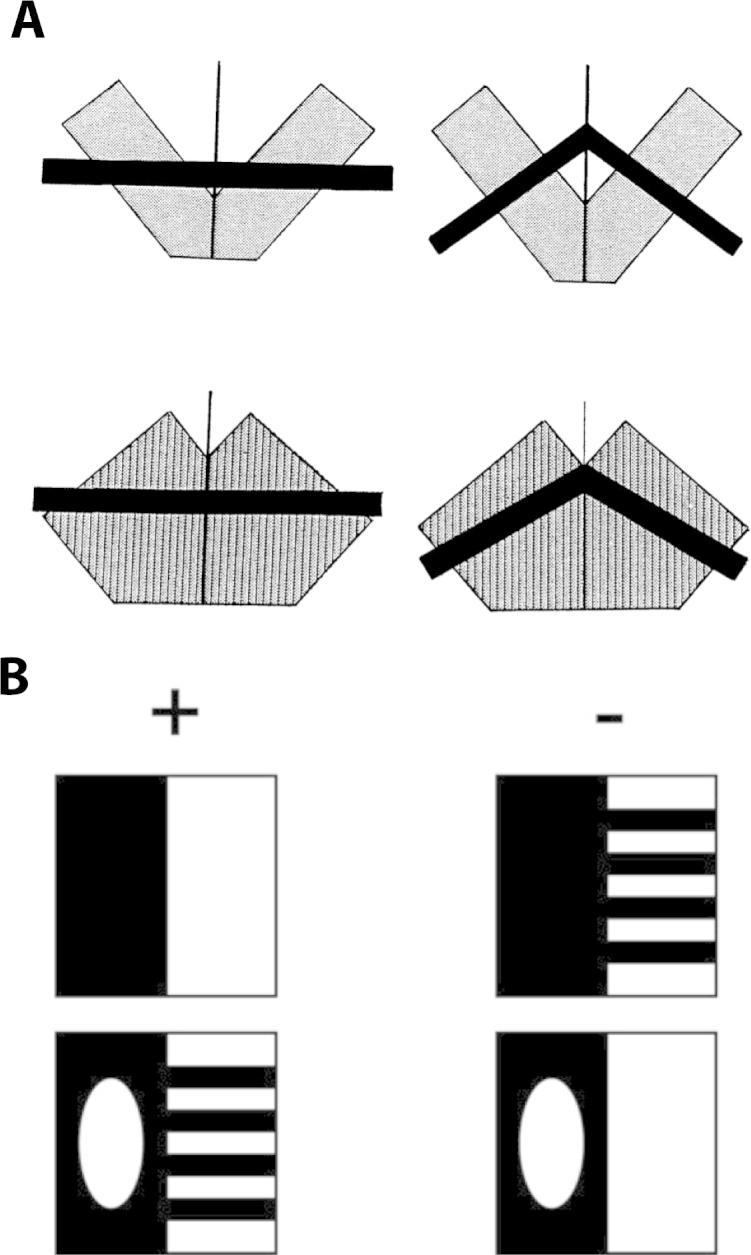
Visual patterns used the test configural discrimination in rats. (A) The stimuli used by Eacott et al. [161], which did not enforce a strictly configural processing strategy, because of the unique features created by the intersection of their constituent elements. (B) The stimuli used by Davies et al. [172], which more properly enforced a configural processing strategy.

The first attempt at training rats in such a task was affected by a possible confound: the constituent elements were superimposed on top of each other to produce the compound patterns, thus creating unique features at their intersections that afforded a nonconfigural solution, in spite of the substantial features’ overlap among the discriminanda (see Fig. 4A) [161]. Therefore, the fact that rats successfully learned this task tells more about their remarkable ability to extract behaviorally relevant visual features among very similar patterns, than about their capability to learn and adopt a strictly configural processing strategy. A later study [172] overcame this limitation by designing compound configural stimuli made of flanking rectangular elements (each bearing a specific visual pattern; see Fig. 4B). Rats successfully learned this task, achieving a performance higher than 70% correct in about 12 training sessions, thus showing the capability to concurrently process multiple constituent parts of visual objects. This conclusion is supported by another study, in which rats were tested in a different kind of configural discrimination, known as transverse patterning [171]. The task consisted of three simultaneous two-alternative forced-choice discriminations (i.e., A+ versus B−, B+ versus C−, and C+ versus A−), with each of the three stimuli (i.e., A, B and C) appearing in two discriminations and being associated with reward in one case (+) but not in the other (−). Similarly to the biconditional discrimination, transverse patterning also requires that subjects build a configural representation of stimulus elements. The fact that rats learned to perform this task with high accuracy (>90% correct) confirms that they can reliably acquire conjunctive representations of visual patterns.

#### 4.3.2 Perceptual grouping in rats

That rats could learn to integrate visual information over multiple locations of the visual field was already a major conclusion of Kreschevsky's experiments [143,144] – rats discriminated discontinuous oriented patterns made of identical constituent elements, by grouping such elements according to their proximity (see Section 3.2 and Fig. 2). These results have been confirmed and extended by more recent studies [148–153], thus further demonstrating the ability of this species to integrate distinct visual features into global percepts. Dodwell [148] was the first to replicate Kreschevsky's findings, showing that they are not the result of a novelty effect, i.e., they cannot be explained by rat preference for the less familiar between two visual patterns. In fact, rats trained to choose a positive column pattern over a negative row pattern, not only preferred a *less* familiar vertical grating over a the training-positive column pattern (as originally shown by Kreschevsky), but also preferred the *more* familiar training-negative row pattern over a continuous horizontal grating. In other words, rats perceived as less negative the originally learned negative, discontinuous pattern, when compared to a novel continuous pattern (the grating) with the same orientation. Still more striking was the finding of another experiment, in which rats trained to choose between a positive diagonal grating (D+) and a negative vertical grating (V−) were later tested in a diagonal (D) vs. horizontal (H) grating comparison. Noticeably, some of the rats, rather than choosing the training-positive stimulus (i.e., D), chose the horizontal grating. That is, they appeared to always choose the less vertical stimulus, which was D+ in the case of the trained V− vs. D+ comparison, but was H, in the case of the D vs. H generalization test. Similar results were found for rats originally trained in a H+ vs. D− comparison and later tested in a D vs. V comparison, with some animals choosing the training-negative stimulus D over V, as if their choice was based on comparing the horizontalness of the stimuli, rather than avoiding a rigidly learned, negative, retinal input pattern.

Dodwell and colleagues further explored the integration of discrete elements into global percepts of verticalness and horizontalness [150], showing that rats can discriminate a column array from a row array, even when the separation between adjacent elements along the horizontal dimension is made very close to their separation along the vertical dimension (thus drastically reducing the strength of the proximity cue) – column and row arrays with a ratio between the largest and the smallest element separation as small as 1.3:1 could still be discriminated by rats with an average performance of ∼74% correct. This is an interesting finding, because it suggests that rats may integrate visual information over large spans of the stimulus arrays, so as to accumulate evidence about what dimension (i.e., horizontal or vertical) has the shorter separation between adjacent pattern elements. However, this study only provides a limited assessment of the dependence of perceptual grouping from the properties of the groping cues, mainly because it lacks in control over important stimulus properties, such as the size and separation, in degrees of visual angle, of the constituent elements of the stimulus arrays. A more recent and rigorous study [151] has found that the largest element separation had to be at least twice the smaller separation for rats to perceive row or column patterns with an average accuracy of at least 75% correct (although later studies by some of the same authors suggest a somewhat smaller separation ratio to attain the same criterion performance [152,153]). Moreover, the strength of the proximity cue that was necessary to produce a reliable perceptual grouping was found to be strongly dependent on the overall density of the visual patterns – given constituent elements of 1.2° of visual angle, the ratio between the larger and the smaller element separation that was necessary to achieve the criterion performance increased from 2:1 to 4:1, when the larger element separation was increased from 5.7° to 16.8° of visual angle (thus making the visual pattern sparser). Interestingly, in this study, not only element proximity but also element alignment was used as a grouping cue, with the elements being perfectly aligned only along one dimension. Although the alignment cue, by itself, failed to produce any perceptual grouping, when it was paired to the proximity cue, it enhanced the grouping produced by the latter – for the sparsest pattern (see above), the ratio between the larger and the smaller element separation that was necessary to achieve criterion decreased to 2.5:1, when the alignment cue was also present. As speculated by the authors, this finding suggests that, in rats, not only proximity but also “pattern regularity influences the perceptual organization of the stimulus” [151], although to a much lesser extent than in humans.

The findings presented in this section, as well as Krechevsky's early investigations on perceptual grouping (see Section 3.2), could potentially be accounted by the tuning properties of rat V1 neurons. As found in other mammalian species, rat primary visual cortex contains neurons acting as orientation-selective filters, spanning the full spectrum of possible orientations [62–66,74]. Many of these oriented filters perform an approximately linear weighted sum of the light intensity patterns falling in their receptive fields. As such, a rat V1 neuron with a given orientation preference (e.g., vertical) would be activated by a discontinuous oriented pattern matching its preferred orientation. Such an activation would be more or less strong, depending on the density of the pattern, with the maximal response achieved for continuous oriented gratings matching the preferred orientation and spatial frequency of the neuron. It is easy to see how certain decision strategies relying on the response strength of these oriented filters could lead (e.g.) to a preference for a novel continuous vertical grating over a previously learned discontinuous column pattern (as originally reported by Krechevsky [143]). For instance, this would be the case, if rat decision strategy consisted in choosing the stimulus that maximally activated the bank of vertically oriented filters (i.e., the grating), rather than choosing the stimulus that evoked a previously learned, template response pattern across the filters’ bank (i.e., the column pattern). In other words, the spatially oriented receptive fields of V1 neurons, when combined with a preference for the patterns that maximize the activity of a given set of neurons, could easily account for rat perceptual grouping. This does not imply that Krechevsky's and Dodwell's findings are trivial, because other decision strategies are possible that would lead to a different behavioral outcome (i.e., a different stimulus preference), given the same pattern of activation in V1 (e.g., a template-matching strategy, as previously mentioned). The behavioral findings described here and in Section 3.2 strongly suggest that, among a set of encoding and decoding strategies, rats prefer to rely on an abstract representation of orientation than a precise matching of stored template patterns. As discussed in Section 5.2.3, this can have profound implications in the assessment of how advanced rat pattern vision is. Finally, it is important to mention that not all Krechevsky's and Dodwell's findings can be easily accounted by the mechanistic arguments discussed above. For instance, the lack of preference for a continuous grating over a discontinuous pattern with the same orientation, in the case a rat was trained to discriminate the discontinuous pattern from an X pattern [143], confirms that the way the V1 representation of a visual pattern is read out (to lead to decision) is not a unique, automatic process. Whether orientation will emerge as the discriminant attribute will strongly depend on task demands. Similarly, Dodwell's finding about the preference for a horizontal grating over a diagonal grating, for some of the rats that were originally trained to prefer a diagonal vs. a vertical grating [148], argues for an idiosyncratic, rat-dependent decoding scheme of V1 response patterns – a scheme that can either weight more the activation of a given subpopulation of neurons (e.g., with diagonal preference), or weight more the inactivation of another neuronal subpopulation (e.g., the cells preferring vertical stimuli).

### 4.4 Are rats truly capable of advanced shape processing?

Overall, the picture emerging from the studies reviewed in the previous sections indicates that rats are capable of a quite advanced processing of visual objects. To summarize, rats have been found able to: (1) tolerate size variations and other transformations in the appearance of previously learned patterns; (2) discriminate visual patterns with matched low-level visual properties; (3) flexibly extract position and shape cues, depending on task demands; (4) learn complex configural visual discriminations; (5) spontaneously process composite visual patterns according to perceptual grouping cues; and (6) extract abstract stimulus dimensions (such as verticalness and horizontalness) from trained patterns. However, many of these findings have been recently challenged by a study of Minini and Jeffery [146], who concluded that rats, when tested in a simultaneous (i.e., a two-alternative forced-choice) discrimination task, do not spontaneously rely on a shape-based recognition strategy, at least when other lower-level visual cues are available to successfully accomplish the task.

**Fig. 5:**
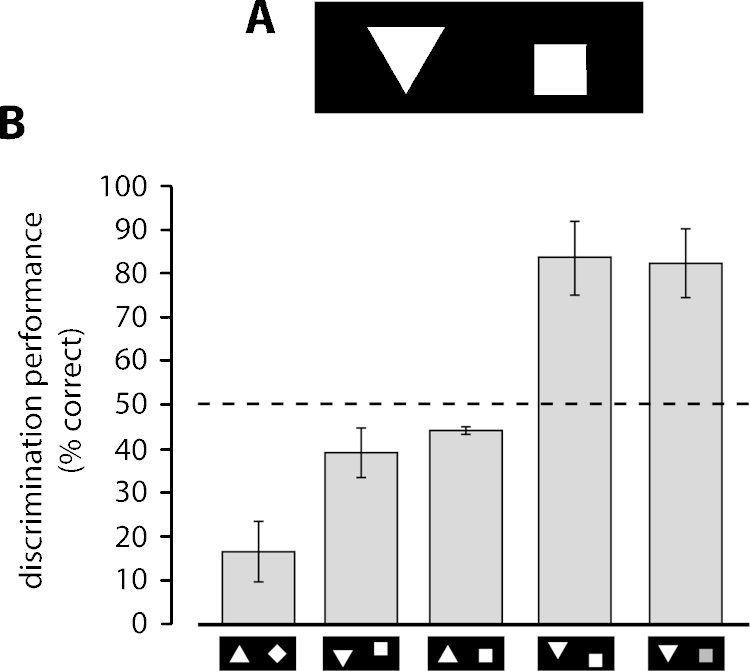
Summary of Minini's and Jeffery's experimental design and results. (A) The triangle vs. square discrimination in which rats were trained in one of Minini's and Jeffery's experiments. (B) Rat discrimination performance for different kinds of manipulations of the originally learned patterns (shown below the abscissa axis). Manipulations altering the relative brightness of the stimuli in the lower hemifield of the stimulus display made rat performance falling to chance (dashed line) or below it (see Section 4.4 for details).

Modified from Minini and Jeffery [146].

Minini and Jeffery carried out two different experiments, each requiring rats to approach a positive target stimulus presented on a touchscreen, while avoiding a simultaneously presented negative stimulus. In the first experiment, rats were trained to discriminate between a white square and a white equilateral triangle (with vertex down) of equal area and equal luminance, shown side by side on the stimulus display (i.e., the touchscreen) against a black background (see Fig. 5A). Following acquisition of the discrimination, rats were tested in a series of transfer trials, where the training shapes were transformed in a variety of ways. Rats failed to generalize to illusory Kanizsa figures and to contrast-reversed stimuli (thus confirming the early observations of Lashley [138] and Field [140]), but succeeded at discriminating the target shapes when these were simultaneously rotated up to 50–60°, thus suggesting some degree of rotation invariance. However, when each shape was rotated or vertically shifted of a different amount compared to the other shape, so as to reverse their brightness relationship in the lower hemifield of the stimulus display, rat performance fell to chance or below it. This was the case when the triangle and the square were rotated, respectively, of 180° and 45°, thus resulting in a comparison between a triangle with vertex up and a diamond (see Fig. 5B, first bar), or when the square only was shifted upward (see Fig. 5B, second bar). A similar trend was found when the difference between the brightens of the two shapes in the lower hemifield was reduced, e.g., when only the triangle was rotated of 180°, so as to bring both shapes to have an horizontal edge in the lower hemifield (see Fig. 5B, third bar). On the other hand, manipulations that did not alter the brightness relationship of the stimuli in the lower hemifield (e.g., when the triangle only was shifted upward; see Fig. 5B, fourth bar) produced no drop in discrimination performance. These led the authors to conclude that rats “used, as the discriminative cue, not the configuration of edges, but differences in the luminance of the two shapes in the lower region of the visual field” [146]. When such a difference reversed (compared to what rats experienced during training with the default stimulus configuration, where the bottom of the square was brighter than the bottom of the triangle; see Fig. 5A), rats failed to discriminate the shapes or even systematically mistook one shape for the other (see Fig. 5B, first bar). However, the authors also noticed that, when the luminance of the square was reduced, so as to match the luminance of the triangle in the lower hemifield, rat recognition performance was not affected (see Fig. 5B, last bar), thus suggesting that “what the rats computed was not absolute luminance per se so much as the area of bright pixels, irrespective of the actual level of brightness” [146].

Obviously, these findings challenge the view that rats are capable of processing visual patters by relying primarily on shape features. As such, they are at odd with many earlier and later findings, presented in the previous and next sections, about visual shape processing in rats, especially those of Gaffan and colleagues [147,160] (see Section 4.2) and Zoccolan and colleagues [118–120] (see Sections 5.1 and 5.2). A way to reconcile these apparently conflicting conclusions is to consider the crucial role that task's structure, constraints and demands likely have in determining the complexity of the visual recognition strategy in rats, as well as in other species (including nonhuman primates; e.g., see [180], discussed in Section 5.2.3). That task's structure could affect the outcome of their experiments was acknowledged by Minini and Jeffery, who pointed out how rat failure to “discover shape” as the diagnostic stimulus dimension “may reflect the constraints of discrimination tasks rather than a limitation in rat vision” [146].

Specifically, in the case of Minini's and Jeffery's experiment, rats were trained in a two-alternative forced-choice discrimination of two geometrical shapes with fixed size, position and brightness (those shown in Fig. 5A). Although during the pre-training procedure the size of the square and triangle undergo some changes in size [146], because of the nature of the two-alternative forced-choice task (see discussion in Section 3.3.1), such size variations were simultaneously applied to both the discriminanda, thus preserving their relative brightness in the lower hemifield. As pointed out by Zoccolan and colleagues, under these circumstances, “computation of lower hemifield luminance was a perfectly ‘valid’ solution to the task at hand (i.e., it was one of many possible strategies for maximizing reward within the context of the experiment)” [118]. By contrast, in Gaffan and Simpson experiments [147], rats were presented in each trial with a different scene comparison (i.e., a constant negative scene paired to a trial-unique variable scene; see Fig. 3A), thus forcing the animals to rely on the specific shape of the objects contained in the scenes, rather than on their low-level properties, such as brightness or area (see Fig. 3C–E). As a final remark, it is worth noticing that, in spite of the lack of task pressure to use shape information, rats in Minini's and Jeffery's first experiment did not exclusively rely on lower-level visual properties to carry out their discrimination. If this was the case, then a manipulation producing a dramatic reversal of the discriminanda relative brightness, such as shifting upward the square, should have produced a full reversal of rat stimulus preference, while, in fact, rat average performance only dropped slightly (and not significantly) below chance (see Fig. 5B, second bar). Instead, such a strong reversal of the stimulus preference was observed only for the vertex-up triangle vs. diamond comparison, i.e., for a manipulation that, in addition to changing the relative brightens of the stimuli, also made the bottom part of the square (now a diamond) very similar to the originally learned vertex-down triangle, and the bottom part of the triangle (now a horizontal edge) very similar to the originally learned square. In other words, it looks like rats did not fully ignore shape – rather, they relied on a strategy that was largely based on a brightness/area comparison, but where shape information (for instance, edge orientation) still played a role.

As previously mentioned, Minini and Jeffery also carried out a second experiment, in which rats were required to discriminate a square from a rectangle, based on the difference in their aspect ratio. The animals eventually learned the task, but only after many sessions (∼70) and only reached a low, albeit significantly higher than chance, performance (∼65% correct). This led the authors to conclude that rats are quite poor at discriminating shapes based on such an abstract metric property as aspect ratio. At the same time, the fact that the rats eventually learned the task suggested to the authors “that rats might be somewhat capable of using aspect ratio, and by implication shape, to solve visual discriminations” [146].

## 5 Invariant visual object recognition and advanced shape processing in rats

Taken together, the studies discussed in Section 4, strongly suggest that rats are capable of processing shape information and recognizing visual objects in spite of some variation in their appearance (e.g., because of size and position changes). However, till very recently, conclusive evidence about invariant recognition and advanced shape processing in rats was lacking, because of four main limitations. First, tolerance of rat recognition to transformations in object appearance had not been systematically tested. That is, none of the studies presented in Section 4 had measured the dependence of rat recognition on the type of transformation an object may undergo, as well as on the magnitude of the transformation along different variation axes (e.g., size, position, in-plane and in-depth rotation, lighting, etc.). The second limitation is that pure generalization of rat recognition to novel object views had never been properly tested, because of the methodological challenge of withholding any feedback to rats about the correctness of their response in generalization/transfer trials (see discussion in Section 3.3.2). Crucially, failure to test pure generalization prevents a robust assessment of what component of rat invariant recognition can be ascribed to a spontaneous, purely perceptual evaluation of visual similarity (i.e., to a true generalization process), and what component, instead, depends on learning arbitrary associative relations among trained object views. The third limitation is that, in spite of the many (often ingenious) stimulus manipulations applied in the studies described in Section 4, no direct assessment of the visual features upon which rats based their recognition was obtained in these investigations. This has prevented a full understanding of the complexity of rat visual processing strategy, leaving open the question of whether this species is truly capable of advanced shape processing (see Section 4.4). Finally, with the exception of perceptual grouping (see Sections 3.2 and 4.3.2), no study had tested rats in those benchmark psychophysics experiments that, in humans, have linked visual perception to fundamental properties of visual cortical processing, such as surround suppression and divisive normalization [181,182].

In Sections 5.1–5.3, I will present recent experimental work that has started to address many of these open issues. As discussed in Section 6, these new findings are very encouraging about the possibility of using the rat as a model to investigate the neuronal substrates of higher-level vision.

### 5.1 Rats are capable of transformation-tolerant (invariant) visual object recognition

**Fig. 6:**
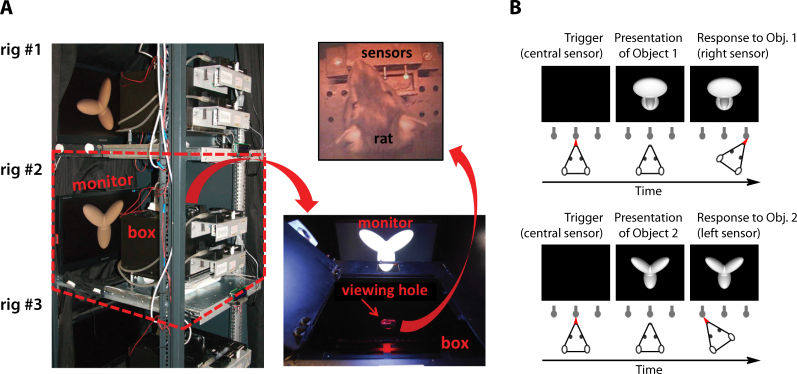
Behavioral rig and experimental design developed by Zoccolan et al. [118]. (A) A picture of the behavioral rig (left), showing three operant boxes, each equipped with monitors for stimulus presentation, computer-controlled pumps for liquid reward delivery, and touch sensors for acquisition of behavioral responses. Rats learned to insert their head through a viewing hole, located in the wall facing the monitor (bottom-right picture), and interact with the array of sensors (top-right picture) to trigger stimulus presentation, report stimulus identity and collect the reward. This rig is currently used in D.D. Cox’ lab (Harvard) and D. Zoccolan's lab (SISSA). A similar high-throughput rig has also been developed by P. Reinagel's lab (UCSD). (B) Schematic of the object discrimination task. After triggering stimulus presentation by licking the central sensor, a rat had to lick either the right or left sensor, depending on object identity.

**Fig. 7:**
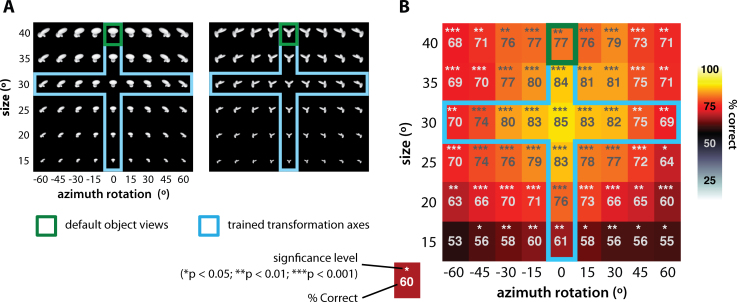
Object conditions and rat group average performance in Zoccolan et al. [118]. (A) The full matrix of size and azimuth-rotation combinations used to test rat invariant recognition in Zoccolan et al. [118]. The green frames show the default object views that rats originally learned. The light blue frames show the transformation axes that rats were trained tolerate, before being bested with the full set of transformations. (B) Rat group average performance for each of the object transformations shown in (A). The percentage of correct trials is both color-coded and reported in each cell, along with its significance. (For interpretation of the references to color in this figure legend, the reader is referred to the web version of this article.)

Modified from Zoccolan et al. [118].

The first systematic investigation of rat invariant recognition was carried out by Zoccolan and colleagues [118] in a study where rats were first trained to discriminate the default views of two visual objects (see Fig. 6B and Fig. 7A, green frames), then required to tolerate some variation in their appearance (see Fig. 7A, light blue frames), and finally tested with many novel transformations of the learned objects (see Fig. 7A, all remaining object conditions). In this study, several rats were trained in parallel, using a high-throughput behavioral rig that consisted of multiple operant boxes, each equipped with a viewing hole, three touch sensors (also acting as juice tubes for liquid reward delivery) and a computer monitor (see Fig. 6A). The rats learned to insert their head into the viewing hole, so as to face the computer monitor (used as the stimulus display), and interact with the sensors to trigger stimulus presentation (central sensor) and report the identity of the visual objects (left and right sensors). This rig, and the stimulus presentation design, allowed a few important methodological advances over earlier behavioral investigations of rat pattern vision. First, they allowed a very tight control over some crucial stimulus properties, such as size, because the stimulus viewing distance was constant and reproducible across trials and sessions. Second, it was possible to carry out a very intensive, fast-pace training, with collection of up to 500 behavioral trials per session per subject. Third, and more importantly, the use of the touch sensors allowed testing rats without resorting to the spatial two-alternative forced-choice procedure used in the large majority of rodent vision studies – in any given trial, a rat was presented with a single stimulus only, and had to report its identity by remembering its association to either the left or right sensor/reward port (see Fig. 6B). Thus, it was possible to overcome the limitation of probing rat invariant recognition with simultaneously presented shapes, which makes it extremely challenging to prevent rats from solving discrimination tasks using lower-level visual features (see discussion in Section 3.3.1 and below). Another major difference with previous studies was that the visual stimuli, rather than being simple geometrical shapes/patterns, were renderings of 3-dimensional object models made of multiple structural parts (see Figs. 6B and7A). This allowed transforming the objects along natural variation axes, such as in-depth rotations and lighting changes, which are impossible to test with 2-dimensional shapes. Therefore, the variety and complexity of the transformations the stimuli underwent in this study were much larger than in previous work.

In [118], rats easily succeeded at discriminating the default views of the visual objects with a >70% correct accuracy, and quickly learned to tolerate size changes between 15° and 40° of visual angle (see Fig. 7A, vertical light blue frames) and in-depth azimuth rotations over a ±60° span (see Fig. 7A, horizontal light blue frames). Following this exposure to size and azimuth changes, rats were tested with a large battery of object conditions, obtained by combining all possible previously trained size and rotation values (i.e., all object conditions shown in Fig. 7A). Crucially, most of these object conditions were new for the rats (i.e., those outside the light blue frames in Fig. 7A). Rat recognition performance was high and significantly larger than chance for virtually all tested transformations (see Fig. 7B), the only substantial drop being observed for the smallest tested size of 15° of visual angle (likely because of rat poor visual acuity). This result shows how rats can tolerate substantial variation in object appearance across very different variation axes and their combination. It also suggests that this ability is likely rooted in a generalization process, based on the perceived similarity between the transformed and the originally learned object views. In fact, given the large number of tested conditions (108), it was unlikely that rats succeeded in the task by learning and memorizing the correct association between each newly presented view and the corresponding reward port.

That rat invariant recognition was the result of pure generalization was also explicitly and rigorously tested, by withholding any feedback to the rats about the correctness of their response for a fraction of the new transformations (approximately 11%, covering a contiguous quadrant of the size-view matrix, with each rat being tested with a different no-feedback quadrant). It is worth noticing that, differently from previous studies, these generalization trials were not just “always rewarded” (a training strategy that, as discussed in Section 3.3.2, can lead to either an overestimation or underestimation of rat recognition accuracy, depending on the choices of an animal on the first few generalization trials). Rather, after a rat made a response in a generalization trial, the stimulus was immediately removed from the display, the trial ended, and the animal was allowed to trigger the next trial right away. The fact that rat recognition performance for these no-feedback conditions was as high as for the (size-matched) feedback conditions (in both cases, it was ∼75% correct), and both were as high as the performance over the originally trained size and azimuth axes, indicates that rats were capable of pure generalization to novel object views. This conclusion was further corroborated by observing that “performance was high and significantly above chance […], even for the very first presentation of each novel stimulus, and remained stable over the course of the experiment” [118]. As a final assessment of rat generalization ability, Zoccolan and colleagues also introduced transformations along two new axes – in-depth elevation rotation and lighting (all these new conditions, 15 per axis, were presented in no-feedback, generalization trials). Performance was above chance for all but one of the novel lighting conditions (with an overall recognition accuracy of ∼75% correct), and all the novel elevation conditions.

Overall, these results led Zoccolan and colleagues to conclude “that rats were able to generalize across a wide range of previously unseen object transformations, including novel combinations of size and in-depth rotations of the learned objects […], new lighting/shading patterns over the objects’ surface […], and also substantial variation in object silhouette […]”[118]. Although this study did not explicitly address what object processing strategy underlay rat invariant recognition, several factors argue in favor of higher-level, shape-based processing, rather than reliance on lower-level visual properties. First, because each view of an object was presented in isolation, a rat could not directly compare it to a similarly transformed view of the other object, as it typically happens in spatial two-alternative forced-choice tasks (see discussion in Section 3.3.1). Rather, the animal had to implicitly compare this view to all other possible transformed views of the other object to succeed in the task. Given the substantial size variations the object underwent, this made many lower-level cues (such as the relative brightness or contrast of the discriminanda) completely unreliable as predictors of object identity. In other words, the use of a lower-level strategy, such as the one reported by Minini and Jeffery [146], was ruled out by the experimental design itself. In addition, Zoccolan and colleagues performed a stimulus analysis showing that, at the level of pixel or Gabor filter representations (simulating, respectively, neuronal representations in retina and primary visual cortex), the average image variation produced by changing the appearance of an object was larger than the average variation between matching views of the two objects. In other words, rat successful recognition of the transformed views of an object could not easily be ascribed to their similarity in a simulated lower-level (i.e., retinal or V1) representation.

#### 5.1.1 Visual priming as a way to assess transformation-tolerant perception of visual objects in rats

The findings of Zoccolan and colleagues [118] provide a robust assessment of rat capability to recognize visual objects in spite of substantial variation in their appearance along a variety of transformation axes. In particular, generalization of rat recognition was rigorously tested using no-feedback trials. Nevertheless, in a follow-up study [119], Zoccolan and collaborators carried out a further investigation of rat generalization abilities, to dissociate more accurately the relative contribution of perceptual similarity and associative learning to rat invariant recognition. The motivation was to overcome a few limitations that still affected the test of generalization in [118]. First, it was possible that rats had learnt to react to the object views presented in generalization trials in a different way than in regular trials, given that, following a response, they had never received neither reward nor punishment. This could have led to a possible underestimation of rat generalization ability. More importantly, rats had received extensive training with some object transformations before and during the test of generalization (since the no-feedback conditions were interleaved with the regular feedback conditions). This raised the question of whether rats actually needed to have such an extensive training with variation in object appearance, or, instead, they were capable of spontaneous generalization to transformed object views, without any previous explicit experience with any object transformation. In other words, it was still unclear what was the baseline tolerance achieved by rats through pure perceptual similarity judgments, and how much improvement over this baseline could be achieved through explicit training/learning. To address these issues, Tafazoli and colleagues [119] adapted to rats a visual priming paradigm – that is, a powerful psychophysics approach that, along with paradigms based on adaptation aftereffects, has been extensively used in humans (and nonhuman primates) to investigate the nature of invariant visual object recognition [183–188]. This method was used to quantify to what extent two different views of an object were perceived as similar by a rat, without providing any feedback to the animal about whether the two views were actually instances of the same object. Such an indirect measurement of perceived similarity allowed a pure assessment of the constancy (or invariance) of object perception in rats, which would have been impossible to achieve otherwise, because of the impact of ongoing learning in standard tests of invariant recognition (see discussion in Sections 3.3.2 and 5.1). In other words, assessing invariant recognition using a priming paradigm prevented the invariance endowed by perceptual constancy to be confounded with the invariance resulting from explicitly learning the associative relations among the different views of an object during training.

**Fig. 8:**
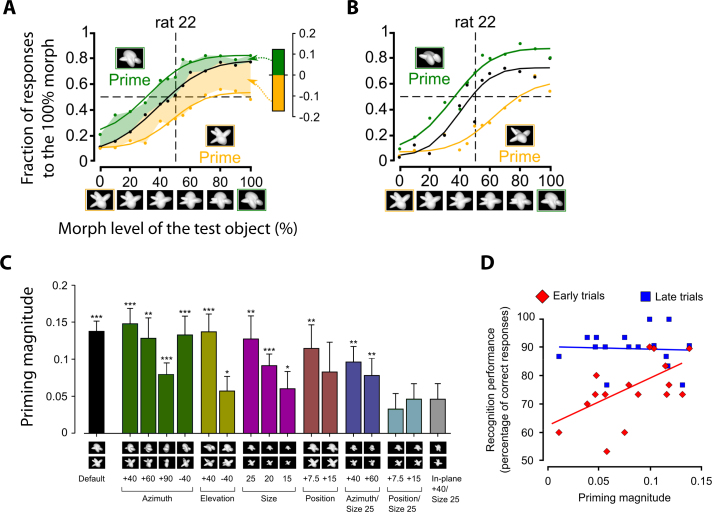
Visual priming produced by default and transformed object views in Tafazoli et al. [119]. (A) Psychometric curves (i.e., fraction of times a morphed object was classified as being more similar to the rightmost prototype, corresponding to the 100% morph level) obtained for an example rat, when no primes were used (black) and when the default views of the 0% (orange) and 100% (green) morph prototypes (shown in the orange/green framed insets) were used as primes. The morph objects are shown below the abscissa (also reporting the morphing level). To quantify the priming magnitude (i.e., to obtain the orange and green bars shown in the inset), the average difference between the psychometric curves obtained in regular and prime trials was computed (orange and green shaded areas). (B) Psychometric curves for the same rat shown in (A), obtained for control trials (black) and prime trials (orange/green), in which 40° elevation-rotated views of the prototypes were used as primes (shown in the insets). (C) Group average priming magnitude (computed as shown in A) produced by all tested priming conditions, i.e., when either the default views (first bar) or the transformed views (all remaining bars) of the object prototypes were used as primes (the tested prototype views are shown below the abscissa; colors refer to transformations of the same kind, but with different magnitude). Asterisks indicate significant priming. (D) Relationship between rat recognition performance and priming magnitude in early (red diamonds) and late (blue squares) trials (data refer to all the 16 tested views of the object prototypes, as shown in C). (For interpretation of the references to color in this figure legend, the reader is referred to the web version of this article.)

Modified from Tafazoli et al. [119].

In Tafazoli's and colleagues’ study, rats were first trained to categorize a continuous shape dimension (or morph line) resulting from blending in different proportions two object prototypes (the prototypes and some of the morphed objects are shown below the abscissa in Fig. 8A and B). The resulting psychometric curves (reporting the fraction of times a morphed object was classified as being more similar to the rightmost prototype, corresponding to the 100% morph level; see black dots/curves in Fig. 8A and B) served as a reference when, in a second phase of the experiment, either prototype was briefly flashed (for ∼50 ms) as a prime, immediately before presentation of a test morphed object. This resulted in either an upward or a downward shift/compression of the psychometric curve, depending on what prototype was used as a prime (see, respectively, the green and orange dots/curves in Fig. 8A and B), showing that rat recognition became biased toward reporting the identity of the prime itself. Critically, this bias was produced not only by the default views of the primes (as shown in Fig. 8A), but also when the primes were transformed along a variety of dimensions (i.e., size, position, in-depth rotation, and their combination) that the animals had never experienced before (Fig. 8B shows, as an example, the priming produced by a 40° in-depth elevation rotation). The priming magnitude was quantified by computing the difference between the psychometric curves obtained in prime and regular trials (see Fig. 8A, orange and green shaded areas) and then averaging across all morph conditions (see Fig. 8A, orange and green bars in the inset). The resulting pattern of average priming magnitudes (see bar plot in Fig. 8C) measured how similar the default and transformed views of the object prototypes were perceived by the rats.

In general, a significant priming was observed for almost all tested transformations, often with a magnitude that was comparable to that produced by the prototypes’ default views (compare the black bar with the other bars in Fig. 8C). This implies that, in most cases, the transformed versions of the object prototypes were perceived as similar to the prototypes’ default views, that is, as instances of the same objects. Not surprisingly though, the degree of perceived similarity depended on the magnitude of the transformation the object underwent (e.g., the larger the azimuth rotation was, the smaller, but still significant, was the resulting priming; see the dark green bars in Fig. 8C). In addition, a few transformations (such as combinations of position and size changes) only produced a modest, not significant priming (see the light blue bars in Fig. 8C). As such, these findings naturally led to further explore the impact that learning could have in enhancing rat invariant recognition, beyond what rats could automatically achieve by relying on the spontaneous, transformation-tolerant perception of visual objects (as estimated by visual priming). To this aim, rat recognition of the transformed prototypes (i.e., those same object views that had been used as primes in the priming experiment) was explicitly tested, by presenting these conditions in regular trials and providing feedback to the animals about the correctness of their responses. When rat responses to the first 10 presentations of the transformed prototypes were considered (early trials), recognition performance was positively and significantly correlated with the previously measured priming magnitude produced by the prototypes’ views (see Fig. 8D, red diamonds). By contrast, when responses collected after rats had become familiar with the transformed prototypes were considered (late trials), rat recognition accuracy had saturated to ceiling values (80–90% correct) and was not correlated any longer with priming magnitude (see Fig. 8D, blue squares).

Overall, these results confirm and extend the conclusions of Zoccolan's and colleagues’ previous study [118] about the ability of rats to generalize their recognition to new appearances of previously encountered objects, without the need of explicit training. At the same time, “the rat visual system, while spontaneously achieving an impressive amount of tolerance along a variety of transformation axes, is far from attaining complete invariance” [119], without the additional contribution provided by explicitly learning the correct associations among different object views. This has led the authors to conclude that “also for rats, as proposed for primates [187–193] and successfully implemented in many leading artificial vision systems [11,14,194,195], transformation-tolerant recognition is achieved by combining the limited (but automatic) tolerance granted by banks of partially-tolerant feature detectors with the fuller tolerance obtained by interpolating between stored representations of multiple, independently learned, object views” [119].

### 5.2 Visual processing strategies underlying rat object vision

Rat ability to recognize visual objects in spite of variation in their appearance strongly suggests that this species processes visual patterns using an advanced, shape-based recognition strategy (according to the definitions provided in Section 1). This conclusion is supported by the choice of stimuli and presentation design of Zoccolan and colleagues [118,119], which makes unlikely a recognition strategy relying upon low-level visual properties, such as overall (or local) brightness, contrast or stimulus surface's area. However, studies based on measurement of recognition performance dot not allow a direct assessment of perceptual strategies. As such, “they cannot tell: (1) whether shape features are truly extracted from the test images; (2) what these features are and how many; and (3) whether they remain stable across the object views the animals face” [120]. To achieve this more complete understanding of rat visual processing it is necessary to apply a class of widely used tools in visual psychophysics, known as *classification image* methods [196]. These methods consist in adding a noise field to the visual patterns a subject has been instructed/trained to recognize, so as to make recognition more or less harder, depending on what parts/regions of the stimulus image are more affected by the noise. By comparing the noise fields leading, respectively, to the correct recognition or to the misidentification of an object, it is possible to uncover the visual features that are diagnostic of that object's identity. Among the many possible implementations of classification images [196], one method, originally proposed by Gosselin and Schyns and known as the *Bubbles* [197], has become very popular as a tool to uncover what visual information a subject extracts in a given classification or discrimination task. Importantly, this method has been successfully applied not only in human studies [197–199], but also in monkey [180,200] and pigeon [201,202] studies of visual perception. More recently, two studies [120,121] exploited this approach to investigate the complexity of rat object recognition strategy.

#### 5.2.1 A flexible, mid-level strategy underlies rat discrimination of simple geometrical shapes

**Fig. 9:**
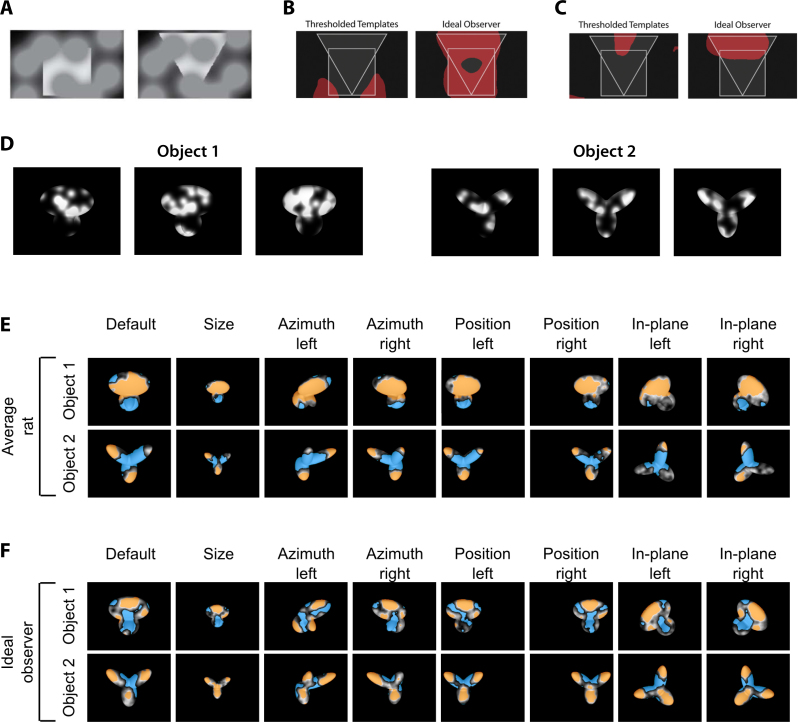
Rat visual object recognition strategy uncovered using the *Bubbles* method. (A) Examples of bubbles masked stimuli used by Vermaercke and Op de Beeck [121]. (B) The regions (in red) that were found to be diagnostic of object identity (named *thresholded behavioral templates*) in Vermaercke's and Op de Beeck's study for rats (left) and for an ideal observer (right). (C) Behavioral templates obtained for the rats (left) and the ideal observer (right), using only trials in which the bottom part of the stimuli was masked. (D) Examples of bubbles masked stimuli used by Alemi-Neissi et al. [120]. (E, F) Saliency maps, showing the patterns of significantly salient (in orange) and anti-salient (in light blue) features obtained for Objects 1 and 2 in the study of Alemi-Neissi and colleagues. (E) and (F) show, respectively, the results for the rats and for a simulated ideal observer. (For interpretation of the references to color in this figure legend, the reader is referred to the web version of this article.)

Vermaercke and Op de Beeck [121] trained rats to discriminate between a triangle and a square, using a spatial two-alternative forced-choice procedure, in which the animals were required “to turn to the screen with the square in order to collect a water reward”. Following acquisition of the discrimination, the authors tested rats with trials in which the discrminanda were partially masked by the same patterns of randomly generated, opaque circular blobs (the bubbles; see Fig. 9A). As described in [197], the bubble masks leading to correct identification were summed and then divided by the sum of all the masks, yielding what the authors called a “behavioral template”, i.e., a map showing, for each pixel over the image plane, the likelihood for the shapes to be correctly discriminated when that pixel was visible through the masks. This map was further processed to extract, according to a statistical criterion, a “thresholded template”, showing what portion of the images was significantly correlated with correct discrimination. Consistent with the findings of Minini and Jeffery [146] (see Section 4.4), this analysis showed that “the animals were mostly using screen positions in the lower part of the display” [121] (see Fig. 9B, left). As previously done in earlier Bubbles studies [197,201,202], Vermaercke and Op de Beeck also compared rat behavioral template to the “optimal” template obtained by simulating an ideal observer. This resulted in a diagnostic region covering most of the stimuli (see Fig. 9B, right), thus showing that “rats used only a small part of the diagnostic information available in the stimuli” [121] – also compared to humans, for which the authors obtained a template that overlapped substantially with the ideal one. However, looking at trials where the bottom part of the stimuli was masked, the authors found that rat performance on these trials was still high (∼70% correct) and larger than chance. When the behavioral template was computed using only these trials, a diagnostic region emerged in the top part of the stimuli (see Fig. 9C, left) that was partially overlapping with the one obtained for the ideal observer (see Fig. 9C, right). Finally, the authors reduced the size of the stimuli by 50%, trained the rats to discriminate these scaled versions of the shapes in spite of translations over the displays (equally applied to both shapes), and finally applied the bubble masks to obtain the behavioral template used during the position-invariant task (which turned out to be quite similar to the one obtained earlier).

Overall, these findings led Vermaercke and Op de Beeck [121] to conclude that “rats are capable of using flexible ‘mid-level’ strategies that include the use of local contrast cues with varying degrees of invariance and context dependence” [121]. In particular, although rat reliance on the bottom part of the stimuli seemed to support Minini's and Jeffery's [146] conclusion about the use of a lower-level strategy (based on comparing brightness in the lower hemifield of the stimulus display; see Section 4.4), the fact that rats successfully exploited differences also on the top part of the stimuli, when forced to do so, indicated that “the behavioral templates of the rats were […] context dependent […], […] demonstrating a flexible strategy that was adapted to the task and challenges at hand” [121]. In other words, Vermaercke's and Op de Beeck's [121] findings, while failing to demonstrate whether rats are truly capable to process shape information, seem to confirm the ability of this species to adapt the complexity of its recognition strategy to task demands, as discussed at length in Section 4.4.

#### 5.2.2 A shape-based, multifeatural processing strategy underlies rat invariant visual object recognition

Alemi-Neissi and colleagues [120] also used the Bubbles method to uncover the perceptual strategy underlying rat recognition of visual objects. Their study differed from the one of Vermaercke and Op de Beeck [121] in many key factors. First, rather than being based on a spatial two-alternative forced-choice task, it used the same stimulus presentation paradigm and behavioral rig developed in Zoccolan's and colleagues’ study [118] (see Section 5.1 and Fig. 6). It also used the same complex, multi-lobed visual objects, which rats were trained to recognize across huge variations in size, in-depth azimuth rotation, in-plane rotation and horizontal translation (i.e., same training procedure as in [118]). Moreover, unique bubble masks were generated and applied to each object view that was tested with the Bubbles method (hence, object- and view-specific saliency maps could be obtained). Finally, the authors’ implementation of the Bubbles method was more similar to the one originally proposed by Gosselin and Schyns [197], with the bubbles being transparent, circular openings, randomly placed over otherwise opaque, black masks (see Fig. 9D). The Bubbles masks were applied to the default object views, as well as to a subset of the transformations used to probe rat invariant recognition: 20° scaled objects, −40° and +20° azimuth rotated objects, ±18° horizontally shifted objects, and ±45° in-plane rotated objects. The bubbles masks were analyzed in the same way of Vermaercke's and Op de Beeck's study [121], so as to yield saliency maps “that measured the correlation between bubbles masks’ transparency values and rat behavioral responses” [120]. For visualization purposes, following Gosselin and Schyns [197], these saliency maps (which are equivalent to Vermaercke's and Op de Beeck's behavioral templates) were converted into “grayscale masks superimposed on the images of the corresponding object views (with the brightness of each pixel indicating the likelihood, for an object view, to be correctly identified when that pixel was visible through the bubbles masks)” [120] (see Fig. 9E and F). A statistical test was used to identify what the authors named “significantly salient and antisalient regions”, i.e., regions over the image plane corresponding “to those objects’ parts that, when visible through the masks, likely led, respectively, to correct identification and misidentification of the object views” [120] (shown, respectively, as orange and light blue patches in Fig. 9E and F) – these are the equivalent of Vermaercke's and Op de Beeck's thresholded templates.

Using this approach, Alemi-Neissi and colleagues [120] uncovered four key aspects of rat recognition strategy. First, rats were found to recognize objects by relying on most or all the distinct structural parts that each object view could afford. For instance, among the two objects in the stimulus set, the one “made of three fully visible, clearly distinct, and approximately equally sized lobes” [120] (Object 2 in Fig. 9E) afforded the larger stimulus complexity and, therefore, “the larger number of ‘perceptual alternatives’ to be used for its correct identification” [120]. Rats made good use of such a structural complexity, by relying, in most cases, on at least two distinct lobes as salient diagnostic features to recognize this object (see Fig. 9E). On the other hand, the presence of a dominant structural feature in the other object (i.e., the large top lobe; see Object 1 in Fig. 9E), limited rat recognition strategy to the detection of such a single feature. A second important finding was that, “for many rats, the recognition strategy was remarkably stable in the face of variation in object appearance. That is, in many cases, the combination of diagnostic structural parts a rat relied upon was the same across all or most of the object views the animal faced” [120]. Another conclusion was that “no trivial low-level strategies (e.g., relying on transformation-preserved diagnostic luminance patches […]) could account for rat invariant recognition behavior” [120]. This can be appreciated by noticing how, in the case of Object 2, the salient lobes were located in both the top and bottom part of the stimulus (see Fig. 9E), thus excluding a preference for the lower hemifield of the display (as reported by Minini and Jeffery [146] and by Vermaercke and Op de Beeck [121]; see, respectively, Sections 4.4 and 5.2.1) and, more importantly, ruling out a strategy based on comparing stimulus luminance in the bottom part of the discriminanda (as concluded by Minini and Jeffery [146]; see Section 4.4). Finally, differently from what found by Vermaercke and Op de Beeck [121] (see Section 5.2.1), “the critical features’ patterns underlying rat recognition strategy closely […] matched those obtained for a simulated ideal observer engaged in the same invariant recognition task” [120] (compare the saliency maps shown in Fig. 9E and F). Taken together, these findings led the authors to conclude that rats are capable of processing “global shape information and make close-to-optimal use of the array of diagnostic features an object is made of […] in a way that is largely tolerant to variation in the appearance of [the] diagnostic object features across a variety of transformation axes” [120].

#### 5.2.3 Reconciling findings about the complexity of visual object processing in rats

Looking at the three most recent studies [120,121,146] addressing the question of how advanced visual object processing is in rats, it could be concluded the following. Minini and Jeffery [146] suggested a natural tendency of rats to rely on the lowest-level global properties that could possibly discriminate the geometrical shapes they tested (namely, local differences in brightness or stimulus surface's area), but did not exclude that rats could learn to discover higher-level diagnostic shape dimensions (see Section 4.4). As speculated in Section 4.4, whether this happens or not is likely to strongly depend on task constraints. Vermaercke and Op de Beeck [121] seemed to confirm this intuition, by showing that rats distinguished between a triangle and a square on the basis of a strategy that could be consistent with what proposed by Minini and Jeffery [146] (see Section 5.2.1). However, the introduction of the bubble masks made the geometrical shapes harder to discriminate, thus pushing the animals to adopt a more flexible strategy, in which the top part of the stimuli was occasionally used as a diagnostic region, in those cases where the bottom part was fully occluded by the masks. However, the poor structural complexity afforded by the square and triangle, combined with the small variety of tested transformations, and, above all, the constraints of the two-alternative forced-choice task (see Section 3.3.1), did not yet allow engaging the full complexity of rat recognition strategy. It took the large set of transformations of the two more structurally complex objects tested by Alemi-Neissi and colleagues [120] (and the application of object-unique bubbles masks to isolated stimuli) to finally reveal that, when properly challenged, rat recognition can be based on a multifeatural, transformation-tolerant processing strategy (see Section 5.2.2). Obviously, these considerations have to be taken as speculations, since no study, so far, has directly tested whether (e.g.) a two-alternative forced-choice task and an isolated-stimulus task would, indeed, bring rats to process the same visual objects using different strategies. Carrying out such comparative studies in a systematic way would be extremely useful to better understand what rats can learn under different circumstances, and, especially, which strategies they tend to use depending on task and stimulus design.

The findings of Alemi-Neissi and colleagues [120] strongly suggest that rats are capable of processing visual objects by extracting and using shape features. They do not imply, however, that such features are the same, or have the same complexity, of those that humans, or higher animals species (such as monkeys), would use. In addition, the saliency regions revealed by the Bubbles method are constrained within the boundaries of the visual stimuli used in the discrimination or classification task. As such, they cannot precisely define how critical is the geometry of a given feature in determining the outcome of the task. For instance, while the saliency maps obtained for Object 2 in the study of Alemi-Neissi and colleagues [120] unequivocally show that rats can integrate visual information from multiple, distinct structural parts of the object (see Fig. 9E, bottom row), those maps do not necessarily imply that rats process/extract the orientation of the lobes. Although rat primary visual cortex contains the neuronal machinery to process orientation [62–66,74], rat object recognition in [120] could be based on detecting a specific spatial arrangement of luminance/contrast patches (i.e., the tips of the lobes) that do not involve extracting the lobes’ orientation. It is an open question whether such a perceptual strategy would qualify as being truly shape-based. According to some authors [121,128], this is not the case, because of the well-demonstrated relevance of oriented edges, boundaries and corners for visual processing in primates [7–10] and brain-inspired models of object vision [11,12,203]. Although this is a very reasonable stance (which questions the usefulness of the rat as a model of higher-level vision), several arguments can be found in favor of the opposite thesis too.

First, many leading computational models of object recognition process the visual input using banks of both oriented and unoriented filters [13] or image fragments [14,15]. Noticeably, some of these models outperform models that include a first stage of processing with oriented filters only (e.g., Gabors) [13], and better predict the response properties of monkey inferotemporal neurons, at both the population and single-cells level [204]. In addition, also some of the models that include an initial bank of oriented Gabors [11,12,203] do not carry out an explicit segmentation of the visual input and, therefore, do not explicitly extract the edges, boundaries and corners that are contained in a scene (neither they estimate their orientation, curvature, etc.). Overall, this suggests that there is no computational reason to consider unoriented contrast patterns as second-rate (not truly shape) features. Rather, it seems that a rich and powerful representation of visual objects must encode both the orientation of their features and the spatial layout of both oriented and unoriented object parts. Therefore, even if rats made use of unoriented contrast patterns only to recognize visual objects (which is highly unlikely, as discussed in the next paragraph), they would still serve as useful models to investigate this aspect of shape processing.

Second, processing of oriented edges/boundaries is well established in monkey primary visual cortex and downstream areas, but is much less documented at the behavioral level. As a matter of fact, those few behavioral studies that have applied the Bubbles method to investigate object vision in monkeys have reached mixed conclusions, with regard to the processing of oriented patterns and corners. In one study, one of the two tested monkeys was found to rely on “image regions that largely lacked spatial structure” [180], when discriminating among natural images (i.e., the monkey relied on small, unoriented patches of the background rather than focus on the foreground object). In another study [200], monkeys recognized the silhouette of a hand using, as salient regions, one or more fingers (similarly to the way rats relied on the lobes of Object 2 in [120]), while humans seemed to rely more consistently on the corner at the conjunction of a pair of fingers. In other words, it is far from obvious that monkeys, in spite of their powerful cortical machinery to process orientation, consistently and preferentially rely on edges and corners, rather than on unoriented feature elements, when engaged in pattern vision. On the other hand, it is unlikely that rats, given the sharp tuning for orientation recently found in rodent primary visual cortex [72,73,129], do not rely at all on the orientation of visual features to discriminate visual patterns, when required to do so by task demands. Indeed, Gaffan's and colleagues’ studies [147,160], with their conclusion that “rats perceived the objects as distinct shapes, not as luminous blobs” [147] (see Section 4.2), as well as rat ability to discriminate the orientation of both continuous and discontinuous patterns [143,150] (see Sections 3.2 and 4.3.2), suggest that this species is fully capable of processing orientation, when needed.

To summarize, there is no strong evidence that a sharp, fundamental distinction exists, between monkeys and rats, insofar as shape processing is concerned. Rather, it is likely that rat and monkey recognition strategies sit along a continuum of shape-processing complexity, where extraction of edges and corners plays an increasingly important role as a function of visual acuity, reaching its peak in primates. Further behavioral studies, perhaps relying on different kinds of classification image approaches (e.g., based on presentation of pure noise fields), and possibly testing both species in the same visual experiment, are necessary to better assess how far apart rats and primates are along this continuum. More importantly, extensive and systematic studies of rat extrastriate visual areas need to be carried out, in order to understand to what extent the coding of visual patterns in these cortical regions is comparable to shape coding along the monkey ventral visual stream (to date, only a single study [205] has addressed this issue).

### 5.3 Rat processing of collinear visual features

**Fig. 10:**
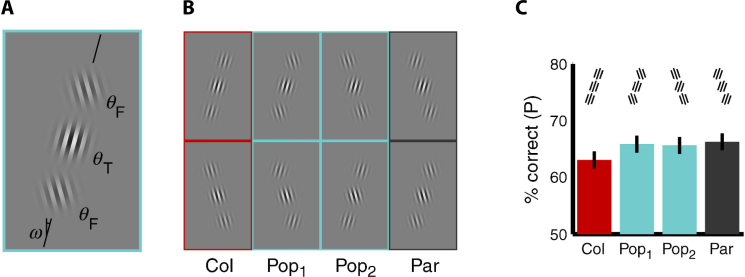
Extracts from Meier et al. [122] study of collinear processing in rats. (A) Example of arrangement of a central target and two flanking gratings. *ϑ_T_* and *ϑ_F_* refer, respectively, to the orientation of the target and flanker gratings, while *ω* indicates the angular position of the flankers (relative to the vertical). (B) The four different kinds of target–flankers configurations tested by Meier et al. (2011). The red frames indicate the collinear conditions, the cyan frames indicate the pop out conditions, while the black frames indicate the parallel conditions (see Section 5.3 for details). (C) The performance on the four flanker conditions for an example rat (the color of the bars matches the color of the frames in B). (For interpretation of the references to color in this figure legend, the reader is referred to the web version of this article.)

Modified from Meier et al. [122].

While the studies of Zoccolan and colleagues [118–120] and Vermaercke and Op de Beeck [121] (and, before them, Minini and Jeffery [146] and Simpson and Gaffan [147]) have investigated rat higher-level vision using rather complex object discrimination tasks, other authors have recently targeted a lower-level aspect of rat pattern vision – the processing of collinearity [122,126,127]. Specifically, Meier and colleagues have tested whether, in rats, detection of a centrally presented, low contrast grating, is affected by the simultaneous presentation of 2 flanking gratings that may or not be collinear with the central one (see Fig. 10A). Addressing this question is very relevant to understand shape processing in rats, since the influence of contrast, spatial frequency, and orientation of nearby stimuli on the perception of an oriented target has been extensively studied in humans, and it has been argued that the way collinear features are processed may strongly affect shape perception (e.g., by “enhanc[ing] salience of relevant features such as continuous contours […] or statistically surprising features” [122]). Moreover, the underlying neuronal mechanism (i.e., divisive normalization of neuronal responses by the summed activity of a nearby pool of neurons) is considered one of the fundamental computations underlying both lower-level and higher-level shape processing [4,181,182].

In Meier's and colleagues’ study [122], the rats were trained to detect a target grating, whose “orientation […] was tilted either clockwise or counterclockwise from vertical by a fixed angle”, while ignoring the presence of two flankers that were placed along a virtual line passing through both the target and the flankers (see Fig. 10A). When the orientation of this line (i.e., the angular position *ω* of the flankers) matched the orientation of both the target (*ϑ*_*T*_) and the flanking gratings (*ϑ*_*F*_), the stimuli were arranged according to a collinear configuration (i.e., *ϑ*_*T*_ = *ϑ*_*F*_ = *ω*; see Fig. 10B, left panels). If the target and flankers had the same orientation, but this did not match the flankers’ angular position (i.e., *ϑ*_*T*_ = *ϑ*_*F*_ = −*ω*), the stimuli were arranged according to what the authors called a “parallel configuration” (see Fig. 10B, right panels). When the target and flankers orientations did not match (i.e., *ϑ*_*T*_ = −*ϑ*_*F*_), so-called “pop out” configurations were obtained (see Fig. 10B, middle panels).

All flanker conditions produced a drop in detection performance, as compared to presentation of the isolated target, but the largest impairment was obtained for the collinear condition (see Fig. 10C, red bar). Noticeably, not only the pop out conditions, but also the parallel condition failed to produce a performance drop as large as the one observed for the collinear condition (in spite of the fact that, in both cases, all gratings had the same orientation; i.e., *ϑ*_*T*_ = *ϑ*_*F*_). That is, the observed target–flankers interaction was sensitive to the feature arrangement of the stimuli, in terms of both their relative orientation and angular position. Another interesting finding was that the drop in detection accuracy was mainly driven by a decrease in hit rate, rather than by an increase of false alarms. In other words, the main influence of the collinear flankers was to cause rats to miss the target more often, thus arguing in favor of a more accentuate suppressive effect of the surrounding patterns in case of collinearity with the central target.

Meier and colleagues [122] interpreted these findings as a “demonstration of pattern sensitivity in [rats], showing that such effects can occur even in species that lack orientation columns”, and suggesting a “specialized processing of oriented image features that can be connected to form a continuous contour” [122]. This conclusion that rat “vision is sensitive to the spatial arrangement of [oriented] features” [122], taken together with evidence about rat invariant recognition [118–120], shape processing [120,121,147,160], and perceptual grouping [148–153], suggests that the rat brain must process visual patterns using computations that are far from trivial. However, as discussed in Section 5.2.3, these computations are not necessarily the same as those implemented by the neuronal circuitry of higher species (e.g., primates). As a demonstration, Meier and Reinagel, in a follow-up study [126], found that flankers have the greatest impact on target detection when they are collinear to the target not only in rats, but also in humans. However, while collinear flankers maximally impair performance in rats, they maximally improve performance in humans. This means that processing of collinear features is different between rats and humans. As the authors nicely summarize, “it is not simply that rats lack pattern-specific processing (sensitivity to higher order configurations or feature conjunctions). Both humans and rats demonstrate a perceptual sensitivity particular to collinear stimuli, but between the species, the sign of the effect is reversed” [126].

## 6 Conclusions and perspectives

Since the early, pioneering investigations in the 30s (see Section 3), through the studies of pattern vision, configural discrimination and perceptual grouping between the 70s and the 90s (see Section 4), till the most recent studies of invariant visual object recognition and shape processing (see Section 5), evidence about higher-level visual processing in rats has been growing considerably. In this review, I tried to provide a unified picture of the status of the knowledge about rat visual perception, by critically presenting most of the behavioral studies concerning rat vision, while emphasizing the theoretical and methodological challenges these studies have tackled. The picture emerging from reviewing this literature is very encouraging, in terms of the potential use of rat models in the study of higher-level visual functions. The rat brain appears to perform rather advanced computations over the visual input, so as to support highly demanding functions, such as transformation-tolerant recognition, multifeatural integration of shape information, configural processing of feature elements, and the like. Given the powerful array of methodological approaches that are available in rats (encompassing imaging [75,103], physiology [206,207], optogenetics [100–102] and neuroanatomy [208,209]), rat studies could shed new light into the neuronal computations underlying visual functions, at the level of circuitry, connectivity, synapses and molecules. For instance, rat studies could tackle a few key issues that are difficult to address in monkeys: (1) the role of excitatory and inhibitory interactions in establishing the response and tuning properties of visual neurons during development and adult life [210–213]; (2) the representation of visual objects and natural scenes by very large neuronal ensembles [214,215], possibly simultaneously recorded across multiple visual areas [205]; (3) the dependence of visual processing from brain-states and behavioral-states [113–115,216]; (4) the integration of visual information with other sensory modalities to support cross-modal perception [217–221].

As it can be appreciated by looking at the literature I cited above, these topics are all actively investigated in the mouse mainly, where cutting-edge experimental approaches (e.g., imaging and optogenetics) are currently easier to apply than in the rat. This raises the question of what rodent species (the mouse or the rat) is (or will be) more suitable to investigate visual processing. Currently, the main limitation of the mouse, as a model of visual functions, is the lack of systematic behavioral studies aimed at probing its visual perceptual abilities. Behavioral tests of mouse vision exist, but they have been applied in the context of (e.g.) learning and memory or spatial navigation studies (see [222] for a review), rather than visual perception studies. As a consequence, while it is known that mice can detect gratings [40,223–225], gauge their orientation [226], and discriminate geometrical and pictorial patterns displayed on touchscreens [175,227], it remains unknown whether they are capable of invariant recognition, shape processing, perceptual grouping, multifeatural integration, etc. Given the similarity between the mouse and the rat visual systems at the anatomical and neurophysiological level [32,35,62,72,112,228], it is possible that no fundamental differences exist between these species in terms of visual capabilities. However, in spite of being more accessible than rats to a variety of experimental approaches, mice could simply be much harder to test in complex visual tasks, as the ones successfully used in rats. This suggests that both rodent species will likely serve as a useful complement to the monkey in the study of visual functions – with mice experiments being more suitable to dissect and manipulate the circuitry underlying lower-level visual processing, and rat experiments being more suitable to investigate the neuronal underpinnings of fairly complex visual behaviors.

This does not imply that rat or, more in general, rodent studies could or should replace investigations in higher animal species (such as nonhuman primates). As pointed out in Section 5.2.3, visual cortical processing in rats could rely on different (although advanced) computations, as compared to primates, and this has certainly shown to be the case for the processing of collinear patterns (see Section 5.3). More in general, while the tuning properties of neurons in rat primary visual cortex display many of the features found in higher species (e.g., orientation tuning [62–66,74]), the overall architecture of the visual cortex is different between rats and primates (e.g., rats lack orientation columns [62,75]). Finally, in an evolutionary perspective, the goal subserved by vision in small mammals is likely different than in higher species [1,34] – more navigation- and predator-detection-oriented in rodents, and more object-oriented in primates. This notion has recently received experimental support by the observation that freely moving rats continuously explore the visual environment through frequent, disconjugate eye movements, in such a way to “provide […] comprehensive overhead surveillance for predator detection” [229] (a surprising finding, given that previous studies in head-restrained rats only reported sporadic and conjugate eye movements [230,231]).

To summarize, two conclusions can be derived from the studies presented in this review. On the one hand, the demonstrated ability of rats to perform unexpectedly advanced visual tasks should not lead to simply equate rodent and primate vision. On the other hand, differences between rat and primate visual behavior (and its underlying neuronal substrates) should not prevent vision scientists from exploiting rats to design and carry out experiments that, at the moment, are precluded in monkeys. In fact, insofar as rats display advanced, vision-based behaviors (which cannot trivially be accounted by lower-level processing strategies), uncovering the neuronal substrates of such behaviors will provide powerful insights into the computations that the brain circuitry can implement to support object vision and other visual functions. In a comparative perspective, these findings will also advance our understanding of how those same computational challenges are solved by more advanced brains.

## Figures and Tables

Panel A is modified from Eacott et al. [161]. Panel B is modified from Davies et al. [172].

Panel B is modified from Alemi-Neissi et al. [120].

Panels (A)–(C) are modified from Vermaercke and Op de Beeck [121]. Panels (D)–(F) are modified from Alemi-Neissi et al. [120].

## References

[1] Cox D.D. (2014). Do we understand high-level vision?. Curr Opin Neurobiol.

[2] Rust N.C., Stocker A.A. (2010). Ambiguity and invariance: two fundamental challenges for visual processing. Curr Opin Neurobiol.

[3] Ullman S. (1996). High level vision.

[4] DiCarlo J.J., Zoccolan D., Rust N.C. (2012). How does the brain solve visual object recognition?. Neuron.

[5] DiCarlo J.J., Cox D.D. (2007). Untangling invariant object recognition. Trends Cogn Sci.

[6] Logothetis N.K., Sheinberg D.L. (1996). Visual object recognition. Ann Rev Neurosci.

[7] Brincat S.L., Connor C.E. (2004). Underlying principles of visual shape selectivity in posterior inferotemporal cortex. Nat Neurosci.

[8] Yamane Y., Carlson E.T., Bowman K.C., Wang Z., Connor C.E. (2008). A neural code for three-dimensional object shape in macaque inferotemporal cortex. Nat Neurosci.

[9] Pasupathy A., Connor C.E. (1999). Responses to contour features in macaque area V4. J Neurophysiol.

[10] Pasupathy A., Connor C.E. (2002). Population coding of shape in area V4. Nat Neurosci.

[11] Riesenhuber M., Poggio T. (1999). Hierarchical models of object recognition in cortex. Nat Neurosci.

[12] Serre T., Oliva A., Poggio T. (2007). A feedforward architecture accounts for rapid categorization. Proc Natl Acad Sci.

[13] Pinto N., Doukhan D., DiCarlo J.J., Cox D.D. (2009). A high-throughput screening approach to discovering good forms of biologically inspired visual representation. PLoS Comput Biol.

[14] Ullman S. (2007). Object recognition and segmentation by a fragment-based hierarchy. Trends Cogn Sci.

[15] Ullman S., Vidal-Naquet M., Sali E. (2002). Visual features of intermediate complexity and their use in classification. Nat Neurosci.

[16] Baldassi C., Alemi-Neissi A., Pagan M., DiCarlo J.J., Zecchina R., Zoccolan D. (2013). Shape similarity, better than semantic membership, accounts for the structure of visual object representations in a population of monkey inferotemporal neurons. PLoS Comput Biol.

[17] Riesenhuber M., Poggio T. (2000). Models of object recognition. Nat Neurosci.

[18] Pinto N., Cox D.D., DiCarlo J.J. (2008). Why is real-world visual object recognition hard?. PLoS Comput Biol.

[19] Biederman I. (1987). Recognition-by-components: a theory of human image understanding. Psychol Rev.

[20] Potter M.C. (1976). Short-term conceptual memory for pictures. J Exp Psychol Hum Learn.

[21] Thorpe S.J., Fize D., Marlot C. (1996). Speed of processing in the human visual system. Nature.

[22] Tanaka K. (1996). Inferotemporal cortex and object vision. Annu Rev Neurosci.

[23] Rolls E.T. (2000). Functions of the primate temporal lobe cortical visual areas in invariant visual object and face recognition. Neuron.

[24] Rousselet G.A., Thorpe S.J., Fabre-Thorpe M. (2004). How parallel is visual processing in the ventral pathway?. Trends Cogn Sci.

[25] Kourtzi Z., Connor C.E. (2011). Neural representations for object perception: structure, category, and adaptive coding. Annu Rev Neurosci.

[26] Hung C.P., Kreiman G., Poggio T., DiCarlo J.J. (2005). Fast readout of object identity from macaque inferior temporal cortex. Science.

[27] Li N., Cox D.D., Zoccolan D., DiCarlo J.J. (2009). What response properties do individual neurons need to underlie position and clutter “invariant” object recognition?. J Neurophysiol.

[28] Rust N.C., DiCarlo J.J. (2010). Selectivity and tolerance (“invariance”) both increase as visual information propagates from cortical area V4 to IT. J Neurosci.

[29] Felleman D.J., Van Essen D.C. (1991). Distributed hierarchical processing in the primate cerebral cortex. Cereb Cortex.

[30] Nassi J.J., Callaway E.M. (2009). Parallel processing strategies of the primate visual system. Nat Rev Neurosci.

[31] Orban G.A. (2008). Higher order visual processing in macaque extrastriate cortex. Physiol Rev.

[32] Wang Q., Gao E., Burkhalter A. (2011). Gateways of ventral and dorsal streams in mouse visual cortex. J Neurosci.

[33] Wang Q., Sporns O., Burkhalter A. (2012). Network analysis of corticocortical connections reveals ventral and dorsal processing streams in mouse visual cortex. J Neurosci.

[34] Dean P., Kolb B., Tees R.C. (1990). Sensory cortex: visual perceptual functions. The cerebral cortex of the rat.

[35] Coogan T.A., Burkhalter A. (1993). Hierarchical organization of areas in rat visual cortex. J Neurosci.

[36] Burn C.C. (2008). What is it like to be a rat? Rat sensory perception and its implications for experimental design and rat welfare. Appl Anim Behav Sci.

[37] Werner J.S., Chalupa L.M. (2004). The visual neurosciences.

[38] Werner J.S., Chalupa L.M. (2014). The new visual neurosciences.

[39] Prusky G.T., Harker K.T., Douglas R.M., Whishaw I.Q. (2002). Variation in visual acuity within pigmented, and between pigmented and albino rat strains. Behav Brain Res.

[40] Prusky G.T., West P.W., Douglas R.M. (2000). Behavioral assessment of visual acuity in mice and rats. Vis Res.

[41] Birch D., Jacobs G.H. (1979). Spatial contrast sensitivity in albino and pigmented rats. Vis Res.

[42] Keller J., Strasburger H., Cerutti D.T., Sabel B.A. (2000). Assessing spatial vision – automated measurement of the contrast-sensitivity function in the hooded rat. J Neurosci Methods.

[43] Lashley K.S. (1930). The mechanisms of vision: III. The comparative visual acuity of pigmented and albino rats. J Genet Psychol.

[44] Wiesenfeld Z., Branchek T. (1976). Refractive state and visual acuity in the hooded rat. Vis Res.

[45] Dean P. (1981). Visual pathways and acuity hooded rats. Behav Brain Res.

[46] Dean P. (1981). Grating detection and visual acuity after lesions of striate cortex in hooded rats. Exp Brain Res.

[47] Campbell F.W., Green D.G. (1965). Optical and retinal factors affecting visual resolution. J Physiol.

[48] Campbell F.W., Gubisch R.W. (1966). Optical quality of the human eye. J Physiol.

[49] Curcio C.A., Sloan K.R., Kalina R.E., Hendrickson A.E. (1990). Human photoreceptor topography. J Comp Neurol.

[50] Hirsch J., Curcio C.A. (1989). The spatial resolution capacity of human foveal retina. Vision Res.

[51] Miller M., Pasik P., Pasik T. (1980). Extrageniculostriate vision in the monkey. VII. Contrast sensitivity functions. J Neurophysiol.

[52] Merigan W.H., Katz L.M. (1990). Spatial resolution across the macaque retina. Vision Res.

[53] De Valois R.L., Morgan H., Snodderly D.M. (1974). Psychophysical studies of monkey Vision-III. Spatial luminance contrast sensitivity tests of macaque and human observers. Vision Res.

[54] Diamond M.E., von Heimendahl M., Knutsen P.M., Kleinfeld D., Ahissar E. (2008). “Where” and “what” in the whisker sensorimotor system. Nat Rev Neurosci.

[55] Diamond M.E., von Heimendahl M., Arabzadeh E. (2008). Whisker-mediated texture discrimination. PLoS Biol.

[56] Diamond M.E., Arabzadeh E. (2013). Whisker sensory system – from receptor to decision. Prog Neurobiol.

[57] Uchida N., Mainen Z.F. (2003). Speed and accuracy of olfactory discrimination in the rat. Nat Neurosci.

[58] Rubin B.D., Katz L.C. (2001). Spatial coding of enantiomers in the rat olfactory bulb. Nat Neurosci.

[59] Mainen Z.F. (2006). Behavioral analysis of olfactory coding and computation in rodents. Curr Opin Neurobiol.

[60] Linster C., Johnson B.A., Morse A., Yue E., Leon M. (2002). Spontaneous versus reinforced olfactory discriminations. J Neurosci.

[61] Wallace D.G., Gorny B., Whishaw I.Q. (2002). Rats can track odors, other rats, and themselves: implications for the study of spatial behavior. Behav Brain Res.

[62] Girman S.V., Sauve Y., Lund R.D. (1999). Receptive field properties of single neurons in rat primary visual cortex. J Neurophysiol.

[63] Burne R.A., Parnavelas J.G., Lin C.S. (1984). Response properties of neurons in the visual cortex of the rat. Exp Brain Res.

[64] Parnavelas J.G., Burne R.A., Lin C.S. (1981). Receptive field properties of neurons in the visual cortex of the rat. Neurosci Lett.

[65] Parnavelas J.G., Burne R.A., Lin C.S. (1983). Distribution and morphology of functionally identified neurons in the visual cortex of the rat. Brain Res.

[66] Shaw C., Yinon U., Auerbach E. (1975). Receptive fields and response properties of neurons in the rat visual cortex. Vision Res.

[67] Heimel J.A., Van Hooser S.D., Nelson S.B. (2005). Laminar organization of response properties in primary visual cortex of the gray squirrel (*Sciurus carolinensis*). J Neurophysiol.

[68] Van Hooser S.D., Heimel J.A., Chung S., Nelson S.B., Toth L.J. (2005). Orientation selectivity without orientation maps in visual cortex of a highly visual mammal. J Neurosci.

[69] Van Hooser S.D., Nelson S.B. (2006). The squirrel as a rodent model of the human visual system. Vis Neurosci.

[70] Dräger U.C. (1975). Receptive fields of single cells and topography in mouse visual cortex. J Comp Neurol.

[71] Métin C., Godement P., Imbert M. (1988). The primary visual cortex in the mouse: receptive field properties and functional organization. Exp Brain Res.

[72] Niell C.M., Stryker M.P. (2008). Highly selective receptive fields in mouse visual cortex. J Neurosci.

[73] Bonin V., Histed M.H., Yurgenson S., Reid R.C. (2011). Local diversity and fine-scale organization of receptive fields in mouse visual cortex. J Neurosci.

[74] Wiesenfeld Z., Kornel E.E. (1975). Receptive fields of single cells in the visual cortex of the hooded rat. Brain Res.

[75] Ohki K., Chung S., Ch’ng Y.H., Kara P., Reid R.C. (2005). Functional imaging with cellular resolution reveals precise micro-architecture in visual cortex. Nature.

[76] Espinosa J.S., Stryker M.P. (2012). Development and plasticity of the primary visual cortex. Neuron.

[77] Berardi N., Pizzorusso T., Maffei L. (2000). Critical periods during sensory development. Curr Opin Neurobiol.

[78] Spolidoro M., Sale A., Berardi N., Maffei L. (2009). Plasticity in the adult brain: lessons from the visual system. Exp Brain Res.

[79] Sale A., Berardi N., Maffei L. (2014). Environment and brain plasticity: towards an endogenous pharmacotherapy. Physiol Rev.

[80] Berardi N., Pizzorusso T., Ratto G.M., Maffei L. (2003). Molecular basis of plasticity in the visual cortex. Trends Neurosci.

[81] Brown M.W., Aggleton J.P. (2001). Recognition memory: what are the roles of the perirhinal cortex and hippocampus?. Nat Rev Neurosci.

[82] Bussey T., Saksida L. (2007). Memory, perception, and the ventral visual-perirhinal-hippocampal stream: thinking outside of the boxes. Hippocampus.

[83] Murray E.A., Bussey T., Saksida L. (2007). Visual perception and memory: a new view of medial temporal lobe function in primates and rodents. Annu Rev Neurosci.

[84] Bussey T., Saksida L. (2005). Object memory and perception in the medial temporal lobe: an alternative approach. Curr Opin Neurobiol.

[85] Squire L.R., Wixted J.T., Clark R.E. (2007). Recognition memory and the medial temporal lobe: a new perspective. Nat Rev Neurosci.

[86] Barnett S.A. (2007). The rat: a study in behavior.

[87] Whishaw I.Q., Mittleman G. (1986). Visits to starts, routes, and places by rats (*Rattus norvegicus*) in swimming pool navigation tasks. J Comp Psychol.

[88] Morris R.G.M. (1981). Spatial localization does not require the presence of local cues. Learn Motiv.

[89] Sutherland R.J., Dyck R.H. (1984). Place navigation by rats in a swimming pool. Can J Psychol Can Psychol.

[90] Schenk F. (1985). Development of place navigation in rats from weaning to puberty. Behav Neural Biol.

[91] Maaswinkel H., Whishaw I.Q. (1999). Homing with locale, taxon, and dead reckoning strategies by foraging rats: sensory hierarchy in spatial navigation. Behav Brain Res.

[92] Zoladek L., Roberts W.A. (1978). The sensory basis of spatial memory in the rat. Anim Learn Behav.

[93] Suzuki S., Augerinos G., Black A.H. (1980). Stimulus control of spatial behavior on the eight-arm maze in rats. Learn Motiv.

[94] O’Keefe J., Speakman A. (1987). Single unit activity in the rat hippocampus during a spatial memory task. Exp Brain Res.

[95] O’Keefe J., Conway D.H. (1978). Hippocampal place units in the freely moving rat: why they fire where they fire. Exp Brain Res.

[96] Muller R.U., Kubie J.L. (1987). The effects of changes in the environment on the spatial firing of hippocampal complex-spike cells. J Neurosci.

[97] Gothard K.M., Skaggs W.E., McNaughton B.L. (1996). Dynamics of mismatch correction in the hippocampal ensemble code for space: interaction between path integration and environmental cues. J Neurosci.

[98] Lee I., Yoganarasimha D., Rao G., Knierim J.J. (2004). Comparison of population coherence of place cells in hippocampal subfields CA1 and CA3. Nature.

[99] Jezek K., Henriksen E.J., Treves A., Moser E.I., Moser M.-B. (2011). Theta-paced flickering between place-cell maps in the hippocampus. Nature.

[100] Deisseroth K. (2011). Optogenetics. Nat Methods.

[101] Fenno L., Yizhar O., Deisseroth K. (2011). The development and application of optogenetics. Annu Rev Neurosci.

[102] Tye K.M., Deisseroth K. (2012). Optogenetic investigation of neural circuits underlying brain disease in animal models. Nat Rev Neurosci.

[103] Greenberg D.S., Houweling A.R., Kerr J.N.D. (2008). Population imaging of ongoing neuronal activity in the visual cortex of awake rats. Nat Neurosci.

[104] Luo L., Callaway E.M., Svoboda K. (2008). Genetic dissection of neural circuits. Neuron.

[105] Abbott A. (2010). Neuroscience: the rat pack. Nat News.

[106] Carandini M., Churchland A.K. (2013). Probing perceptual decisions in rodents. Nat Neurosci.

[107] Brunton B.W., Botvinick M.M., Brody C.D. (2013). Rats and humans can optimally accumulate evidence for decision-making. Science.

[108] Murphy R.A., Mondragón E., Murphy V.A. (2008). Rule learning by rats. Science.

[109] Fassihi A., Akrami A., Esmaeili V., Diamond M.E. (2014). Tactile perception and working memory in rats and humans. Proc Natl Acad Sci.

[110] Andermann M.L., Kerlin A.M., Roumis D.K., Glickfeld L.L., Reid R.C. (2011). Functional specialization of mouse higher visual cortical areas. Neuron.

[111] Marshel J.H., Garrett M.E., Nauhaus I., Callaway E.M. (2011). Functional specialization of seven mouse visual cortical areas. Neuron.

[112] Gao E., DeAngelis G.C., Burkhalter A. (2010). Parallel input channels to mouse primary visual cortex. J Neurosci.

[113] Niell C.M., Stryker M.P. (2010). Modulation of visual responses by behavioral state in mouse visual cortex. Neuron.

[114] Ayaz A., Saleem A.B., Schölvinck M.L., Carandini M. (2013). Locomotion controls spatial integration in mouse visual cortex. Curr Biol.

[115] Saleem A.B., Ayaz A., Jeffery K.J., Harris K.D., Carandini M. (2013). Integration of visual motion and locomotion in mouse visual cortex. Nat Neurosci.

[116] Bhattacharjee Y. (2012). A vision of how mouse vision can reveal consciousness’ secrets. Science.

[117] Koch C., Reid R.C. (2012). Neuroscience: observatories of the mind. Nature.

[118] Zoccolan D., Oertelt N., DiCarlo J.J., Cox D.D. (2009). A rodent model for the study of invariant visual object recognition. Proc Natl Acad Sci USA.

[119] Tafazoli S., Di Filippo A., Zoccolan D. (2012). Transformation-tolerant object recognition in rats revealed by visual priming. J Neurosci.

[120] Alemi-Neissi A., Rosselli F.B., Zoccolan D. (2013). Multifeatural shape processing in rats engaged in invariant visual object recognition. J Neurosci.

[121] Vermaercke B., Op de Beeck H.P. (2012). A multivariate approach reveals the behavioral templates underlying visual discrimination in rats. Curr Biol.

[122] Meier P., Flister E., Reinagel P. (2011). Collinear features impair visual detection by rats. J Vis.

[123] Brooks D.I., Ng K.H., Buss E.W., Marshall A.T., Freeman J.H., Wasserman E.A. (2013). Categorization of photographic images by rats using shape-based image dimensions. J Exp Psychol Anim Behav Process.

[124] Petruno S.K., Clark R.E., Reinagel P. (2013). Evidence that primary visual cortex is required for image, orientation, and motion discrimination by rats. PLOS ONE.

[125] Reinagel P. (2013). Speed and accuracy of visual motion discrimination by rats. PLOS ONE.

[126] Meier P.M., Reinagel P. (2013). Rats and humans differ in processing collinear visual features. Front Neural Circuits.

[127] Meier P., Reinagel P. (2011). Rat performance on visual detection task modeled with divisive normalization and adaptive decision thresholds. J Vis.

[128] Vinken K., Vermaercke B., de Beeck H.P.O. (2014). Visual categorization of natural movies by rats. J Neurosci.

[129] Huberman A.D., Niell C.M. (2011). What can mice tell us about how vision works?. Trends Neurosci.

[130] Niell C.M. (2011). Exploring the next frontier of mouse vision. Neuron.

[131] Niell C.M. (2013). Vision: more than expected in the early visual system. Curr Biol.

[132] Lashley K.S. (1912). Visual discrimination of size and form in the albino rat. J Anim Behav.

[133] Munn N.L. (1929). Concerning visual form discrimination in the white rat. Pedagog Semin J Genet Psychol.

[134] Munn N.L. (1930). Visual pattern discrimination in the white rat. J Comp Psychol.

[135] Fields P.E. (1929). The white rats’ use of visual stimuli in the discrimination of geometrical figures. J Comp Psychol.

[136] Fields P.E. (1928). Form discrimination in the white rat. J Comp Psychol.

[137] Lashley K.S. (1930). The mechanisms of vison. I. A method for rapid analysis of pattern-vision in the rat. J Genet Psychol.

[138] Lashley K.S. (1938). The mechanisms of vision: XV. Preliminary studies of the rat's capacity for detail vision. J Gen Psychol.

[139] Munn N.L. (1930). A note on Lashley's method for studying vision in the rat. Pedagog Semin J Genet Psychol.

[140] Fields P.E. (1932). Studies in concept formation. I. The development of the concept of triangularity by the white rat. Comp Psychol Monogr.

[141] Fields P.E. (1935). Studies in concept formation. II. A new multiple stimulus jumping apparatus for visual figure discrimination. J Comp Psychol.

[142] Hebb D.O. (1937). The innate organization of visual activity: I. Perception of figures by rats reared in total darkness. Pedagog Semin J Genet Psychol.

[143] Krechevsky I. (1938). An experimental investigation of the principle of proximity in the visual perception of the rat. J Exp Psychol.

[144] Krechevsky I. (1938). A note on the perception of linear Gestalten in the rat. Pedagog Semin J Genet Psychol.

[145] Fields P.E. (1936). Studies in concept formation. III. A note on the retention of visual figure discriminations. J Comp Psychol.

[146] Minini L., Jeffery K.J. (2006). Do rats use shape to solve “shape discriminations”?. Learn Mem.

[147] Simpson E.L., Gaffan E.A. (1999). Scene and object vision in rats. Q J Exp Psychol B.

[148] Dodwell P.C. (1970). Anomalous transfer effects after pattern discrimination training in rats and squirrels. J Comp Physiol Psychol.

[149] Dodwell P.C., Niemi R.R., Ferguson H.B. (1976). Anomalous transfer in rats: a “macropattern” phenomenon. Bull Psychon Soc.

[150] Dodwell P.C., Ferguson H.B., Niemi R.R. (1976). Anomalous transfer in rats: pattern element separation and discriminability. Bull Psychon Soc.

[151] Kurylo D.D., Van Nest J., Knepper B. (1997). Characteristics of perceptual grouping in rats. J Comp Psychol.

[152] Kurylo D.D., Gazes Y. (2008). Effects of ketamine on perceptual grouping in rats. Physiol Behav.

[153] Kurylo D.D. (2008). Effects of visual cortex lesions on perceptual grouping in rats. Behav Brain Res.

[154] Sutherland N.S. (1961). Visual discrimination of horizontal and vertical rectangles by rats on a new discrimination training apparatus. Q J Exp Psychol.

[155] Sutherland N.S., Carr A.E., Mackintosh J.A. (1962). Visual discrimination of open and closed shapes by Rats. I. Training. Q J Exp Psychol.

[156] Sutherland N.S., Carr A.E. (1962). Visual discrimination of open and closed shapes by Rats. II. Transfer tests. Q J Exp Psychol.

[157] Sutherland N.S., Williams C. (1969). Discrimination of checkerboard patterns by rats. Q J Exp Psychol.

[158] Gaffan E.A., Eacott M.J. (1995). A computer-controlled maze environment for testing visual memory in the rat. J Neurosci Methods.

[159] Gaffan E.A., Woolmore A.L. (1996). Complex visual learning by rats. Learn Motiv.

[160] Gaffan E., Eacott M., Simpson E. (2000). Perirhinal cortex ablation in rats selectively impairs object identification in a simultaneous visual comparison task. Behav Neurosci.

[161] Eacott M.J., Machin P.E., Gaffan E.A. (2001). Elemental and configural visual discrimination learning following lesions to perirhinal cortex in the rat. Behav Brain Res.

[162] Gaffan E.A., Bannerman D.M., Warburton E.C., Aggleton J.P. (2001). Rats’ processing of visual scenes: effects of lesions to fornix, anterior thalamus, mamillary nuclei or the retrohippocampal region. Behav Brain Res.

[163] Eacott M.J., Norman G., Gaffan E.A. (2003). The role of perirhinal cortex in visual discrimination learning for visual secondary reinforcement in rats. Behav Neurosci.

[164] Gaffan E.A., Healey A.N., Eacott M.J. (2004). Objects and positions in visual scenes: effects of perirhinal and postrhinal cortex lesions in the rat. Behav Neurosci.

[165] Winters B.D., Forwood S., Cowell R.A., Saksida L., Bussey T. (2004). Double dissociation between the effects of peri-postrhinal cortex and hippocampal lesions on tests of object recognition and spatial memory: heterogeneity of function within the temporal lobe. J Neurosci.

[166] Winters B.D., Bussey T. (2005). Transient inactivation of perirhinal cortex disrupts encoding, retrieval, and consolidation of object recognition memory. J Neurosci.

[167] Winters B.D., Bussey T. (2005). Glutamate receptors in perirhinal cortex mediate encoding, retrieval, and consolidation of object recognition memory. J Neurosci.

[168] Huston A.E., Aggleton J.P. (1987). The effects of cholinergic drugs upon recognition memory in rats. Q J Exp Psychol Sect B.

[169] Astur R.S., Klein R.L., Mumby D.G., Protz D.K., Sutherland R.J., Martin G.M. (2002). A role for olfaction in object recognition by normal and hippocampal-damaged rats. Neurobiol Learn Mem.

[170] Prusky G.T., Douglas R.M., Nelson L., Shabanpoor A., Sutherland R.J. (2004). Visual memory task for rats reveals an essential role for hippocampus and perirhinal cortex. Proc Natl Acad Sci USA.

[171] Driscoll I., Howard S.R., Prusky G.T., Rudy J.W., Sutherland R.J. (2005). Seahorse wins all races: hippocampus participates in both linear and non-linear visual discrimination learning. Behav Brain Res.

[172] Davies M., Machin P., Sanderson D., Pearce J., Aggleton J. (2007). Neurotoxic lesions of the rat perirhinal and postrhinal cortices and their impact on biconditional visual discrimination tasks. Behav Brain Res.

[173] Bussey T., Padain T., Skillings E., Winters B., Morton A., Saksida L. (2008). The touchscreen cognitive testing method for rodents: how to get the best out of your rat. Learn Mem.

[174] Cook R.G., Geller A.I., Zhang G.-R., Gowda R. (2004). Touchscreen-enhanced visual learning in rats. Behav Res Methods Instrum Comput.

[175] Horner A.E., Heath C.J., Hvoslef-Eide M., Kent B.A., Kim C.H., Nilsson S.R.O. (2013). The touchscreen operant platform for testing learning and memory in rats and mice. Nat Protoc.

[176] Bussey T.J., Muir J.L., Everitt B.J., Robbins T.W. (1997). Triple dissociation of anterior cingulate, posterior cingulate, and medial frontal cortices on visual discrimination tasks using a touchscreen testing procedure for the rat. Behav Neurosci.

[177] Bussey T.J., Everitt B.J., Robbins T.W. (1997). Dissociable effects of cingulate and medial frontal cortex lesions on stimulus-reward learning using a novel Pavlovian autoshaping procedure for the rat: implications for the neurobiology of emotion. Behav Neurosci.

[178] Baker C.I., Behrmann M., Olson C.R. (2002). Impact of learning on representation of parts and wholes in monkey inferotemporal cortex. Nat Neurosci.

[179] Cox D.D., DiCarlo J.J. (2008). Does learned shape selectivity in inferior temporal cortex automatically generalize across retinal position?. J Neurosci.

[180] Nielsen K.J., Logothetis N.K., Rainer G. (2006). Discrimination strategies of humans and rhesus monkeys for complex visual displays. Curr Biol.

[181] Carandini M., Heeger D.J. (2012). Normalization as a canonical neural computation. Nat Rev Neurosci.

[182] Schwartz O., Simoncelli E.P. (2001). Natural signal statistics and sensory gain control. Nat Neurosci.

[183] Biederman I., Cooper E.E. (1992). Size invariance in visual object priming. J Exp Psychol Hum Percept Perform.

[184] Suzuki S., Cavanagh P. (1998). A shape-contrast effect for briefly presented stimuli. J Exp Psychol Hum Percept Perform.

[185] Bar M., Biederman I. (1998). Subliminal visual priming. Psychol Sci.

[186] Leopold D.A., O’Toole A.J., Vetter T., Blanz V. (2001). Prototype-referenced shape encoding revealed by high-level aftereffects. Nat Neurosci.

[187] Afraz S.-R., Cavanagh P. (2008). Retinotopy of the face aftereffect. Vis Res.

[188] Kravitz D.J., Kriegeskorte N., Baker C.I. (2010). High-level visual object representations are constrained by position. Cereb Cortex.

[189] Lawson R. (1999). Achieving visual object constancy across plane rotation and depth rotation. Acta Psychol (Amst).

[190] Logothetis N.K., Pauls J., Bulthoff H.H., Poggio T. (1994). View-dependent object recognition by monkeys. Curr Biol.

[191] Bülthoff H.H., Edelman S.Y., Tarr M.J. (1995). How are three-dimensional objects represented in the brain?. Cereb Cortex (New York, N.Y.: 1991).

[192] Tarr M.J., Bülthoff H.H. (1998). Image-based object recognition in man, monkey and machine. Cognition.

[193] Kravitz D.J., Vinson L.D., Baker C.I. (2008). How position dependent is visual object recognition?. Trends Cogn Sci.

[194] Poggio T., Edelman S. (1990). A network that learns to recognize three-dimensional objects. Nature.

[195] Ullman S., Soloviev S. (1999). Computation of pattern invariance in brain-like structures. Neural Netw.

[196] Murray R.F. (2011). Classification images: a review. J Vis.

[197] Gosselin F., Schyns P.G. (2001). Bubbles: a technique to reveal the use of information in recognition tasks. Vis Res.

[198] Schyns P., Bonnar L., Gosselin F. (2002). Show me the features! Understanding recognition from the use of visual information. Psychol Sci.

[199] Butler S., Blais C., Gosselin F., Bub D., Fiset D. (2010). Recognizing famous people. Atten Percept Psychophys.

[200] Nielsen K.J., Logothetis N.K., Rainer G. (2008). Object features used by humans and monkeys to identify rotated shapes. J Vis.

[201] Gibson B.M., Wasserman E.A., Gosselin F., Schyns P.G. (2005). Applying bubbles to localize features that control pigeons’ visual discrimination behavior. J Exp Psychol Anim Behav Process.

[202] Gibson B.M., Lazareva O.F., Gosselin F., Schyns P.G., Wasserman E.A. (2007). Nonaccidental properties underlie shape recognition in mammalian and nonmammalian vision. Curr Biol.

[203] Serre T., Wolf L., Bileschi S., Riesenhuber M., Poggio T. (2007). Robust object recognition with cortex-like mechanisms. IEEE Trans Pattern Anal Mach Intell.

[204] Yamins D.L.K., Hong H., Cadieu C.F., Solomon E.A., Seibert D., DiCarlo J.J. (2014). Performance-optimized hierarchical models predict neural responses in higher visual cortex. Proc Natl Acad Sci.

[205] Vermaercke B., Gerich F.J., Ytebrouck E., Arckens L., de Beeck H.P.O., den Bergh G.V. (2014). Functional specialization in rat occipital and temporal visual cortex. J Neurophysiol.

[206] Lee A.K., Manns I.D., Sakmann B., Brecht M. (2006). Whole-cell recordings in freely moving rats. Neuron.

[207] Margrie T.W., Brecht M., Sakmann B. (2002). In vivo, low-resistance, whole-cell recordings from neurons in the anaesthetized and awake mammalian brain. Pflugers Arch.

[208] Egger R., Narayanan R.T., Helmstaedter M., de Kock C.P.J., Oberlaender M. (2012). 3D reconstruction and standardization of the rat vibrissal cortex for precise registration of single neuron morphology. PLoS Comput Biol.

[209] Meyer H.S., Egger R., Guest J.M., Foerster R., Reissl S., Oberlaender M. (2013). Cellular organization of cortical barrel columns is whisker-specific. Proc Natl Acad Sci.

[210] Pecka M., Han Y., Sader E., Mrsic-Flogel T.D. (2014). Experience-dependent specialization of receptive field surround for selective coding of natural scenes. Neuron.

[211] Kimura R., Safari M.-S., Mirnajafi-Zadeh J., Kimura R., Ebina T., Yanagawa Y. (2014). Curtailing effect of awakening on visual responses of cortical neurons by cholinergic activation of inhibitory circuits. J Neurosci.

[212] Vaiceliunaite A., Erisken S., Franzen F., Katzner S., Busse L. (2013). Spatial integration in mouse primary visual cortex. J Neurophysiol.

[213] Adesnik H., Bruns W., Taniguchi H., Huang Z.J., Scanziani M. (2012). A neural circuit for spatial summation in visual cortex. Nature.

[214] Froudarakis E., Berens P., Ecker A.S., Cotton R.J., Sinz F.H., Yatsenko D. (2014). Population code in mouse V1 facilitates readout of natural scenes through increased sparseness. Nat Neurosci.

[215] Miller J.K., Ayzenshtat I., Carrillo-Reid L., Yuste R. (2014). Visual stimuli recruit intrinsically generated cortical ensembles. Proc Natl Acad Sci.

[216] Bennett C., Arroyo S., Hestrin S. (2013). Subthreshold mechanisms underlying state-dependent modulation of visual responses. Neuron.

[217] Raposo D., Sheppard J.P., Schrater P.R., Churchland A.K. (2012). Multisensory decision-making in rats and humans. J Neurosci.

[218] Sheppard J.P., Raposo D., Churchland A.K. (2013). Dynamic weighting of multisensory stimuli shapes decision-making in rats and humans. J Vis.

[219] Iurilli G., Ghezzi D., Olcese U., Lassi G., Nazzaro C., Tonini R. (2012). Sound-driven synaptic inhibition in primary visual cortex. Neuron.

[220] Sieben K., Röder B., Hanganu-Opatz I.L. (2013). Oscillatory entrainment of primary somatosensory cortex encodes visual control of tactile processing. J Neurosci.

[221] Vasconcelos N., Pantoja J., Belchior H., Caixeta F.V., Faber J., Freire M.A.M. (2011). Cross-modal responses in the primary visual cortex encode complex objects and correlate with tactile discrimination. Proc Natl Acad Sci.

[222] Pinto L.H., Enroth-Cugell C. (2000). Tests of the mouse visual system. Mamm Genome.

[223] Prusky G.T., Douglas R.M. (2004). Characterization of mouse cortical spatial vision. Vis Res.

[224] Histed M.H., Carvalho L.A., Maunsell J.H.R. (2012). Psychophysical measurement of contrast sensitivity in the behaving mouse. J Neurophysiol.

[225] Busse L., Ayaz A., Dhruv N.T., Katzner S., Saleem A.B., Schölvinck M.L. (2011). The detection of visual contrast in the behaving mouse. J Neurosci.

[226] Wong A.A., Brown R.E. (2006). Visual detection, pattern discrimination and visual acuity in 14 strains of mice. Genes Brain Behav.

[227] Nithianantharajah J., Komiyama N.H., McKechanie A., Johnstone M., Blackwood D.H., Clair D.S. (2013). Synaptic scaffold evolution generated components of vertebrate cognitive complexity. Nat Neurosci.

[228] Espinoza S.G., Thomas H.C. (1983). Retinotopic organization of striate and extrastriate visual cortex in the hooded rat. Brain Res.

[229] Wallace D.J., Greenberg D.S., Sawinski J., Rulla S., Notaro G., Kerr J.N.D. (2013). Rats maintain an overhead binocular field at the expense of constant fusion. Nature.

[230] Zoccolan D., Graham B.J., Cox D.D. (2010). A self-calibrating, camera-based eye tracker for the recording of rodent eye movements. Front Neurosci.

[231] Chelazzi L., Rossi F., Tempia F., Ghirardi M., Strata P. (1989). Saccadic eye movements and gaze holding in the head-restrained pigmented rat. Eur J Neurosci.

